# Protective Microglial Subset in Development, Aging, and Disease: Lessons From Transcriptomic Studies

**DOI:** 10.3389/fimmu.2020.00430

**Published:** 2020-04-03

**Authors:** Anouk Benmamar-Badel, Trevor Owens, Agnieszka Wlodarczyk

**Affiliations:** ^1^Department of Neurobiology Research, Institute for Molecular Medicine, University of Southern Denmark, Odense, Denmark; ^2^BRIDGE, Brain Research - Inter-Disciplinary Guided Excellence, Odense, Denmark; ^3^Department of Neurology, Slagelse Hospital, Institute of Regional Health Research, Slagelse, Denmark

**Keywords:** microglia, CD11c microglia, heterogeneity, CD11c, transcriptomics, subset, DAM, single cell

## Abstract

Microglial heterogeneity has been the topic of much discussion in the scientific community. Elucidation of their plasticity and adaptability to disease states triggered early efforts to characterize microglial subsets. Over time, their phenotypes, and later on their homeostatic signature, were revealed, through the use of increasingly advanced transcriptomic techniques. Recently, an increasing number of these “microglial signatures” have been reported in various homeostatic and disease contexts. Remarkably, many of these states show similar overlapping microglial gene expression patterns, both in homeostasis and in disease or injury. In this review, we integrate information from these studies, and we propose a unique subset, for which we introduce a core signature, based on our own research and reports from the literature. We describe that this subset is found in development and in normal aging as well as in diverse diseases. We discuss the functions of this subset as well as how it is induced.

## Introduction

The term “microglia” was brought to the scientific community's attention a century ago with its first use by Pio del Rio-Hortega (1), who strived to distinguish them from oligodendrocytes. His early work also highlighted their phagocytic ability, as well as their potential to undergo morphological changes. This early description led the community to consider microglial cells as a homogeneous population, even though the first description of a microglial subset (“satellite microglia”) appeared as early as 1919 (1).

Microglia originate from yolk-sac progenitors that start migrating toward the fetus around mid-pregnancy. These progenitors reach the embryonic brain around embryonic day (E) 9.5–E10.5 (2, 3) until the formation of the blood–brain barrier around E13.5–E14.5 in the mouse, and between the 4th gestational week to the 24th gestational week in the human (4, 5). As such, they are among the first cells to colonize the developing brain, and they participate in central nervous system (CNS) development. For instance, they contribute to refine brain wiring through enhancing both synapse formation (6, 7) and elimination (8, 9), they modulate axonal growth (10, 11), they secrete factors promoting neuronal progenitors survival (12) helping with neuronal positioning (11, 13), and they participate in the clearance of live and apoptotic cells during development (14). Microglia also take on physiological functions in the adult CNS, as they constantly sense their immediate environment, in a so-called “never-resting state” (15, 16). Our knowledge of microglial physiology and process motility relies heavily on studies in anesthetized animals. Understanding of microglial functions in the steady state is challenged by a recent study showing that microglial process motility and morphology are affected by the wakefulness state of mice (17). Aside from this surveillance immune function, they are also fundamental for regulation of social behavior, learning, and memory, as these functions are impaired upon their depletion and restored after repopulation (18). Microglial roles in injury and disease contexts have been investigated extensively, with new advances contributing to deepen our understanding of Microglia and their effect on other glial cells [reviewed in Greenhalgh et al. (19)].

These physiological functions advanced our view of microglia, from being initially thought of as exclusively sentinel cells reacting in the context of injury. This dated view on microglia led to the superposition of macrophage M1/M2 phenotypes onto them (20), which was an early attempt to grasp the extent of microglial diversity. This classification is however mostly obsolete nowadays, as it was proved to be simplistic and disconnected from *in vivo* reality (21).

Indeed, the variety of functions microglia take on in space, time, and health states along with reports of sex differences in microglial function have led the community to infer a greater microglial heterogeneity than initially thought. With the progress of technology, investigating such diversity has become possible, notably through the development of high-throughput techniques such as mass cytometry and with the recent advances in transcriptomic studies with single-cell RNA-sequencing (RNA-seq). These technologies allowed the identification of microglial signatures linked to their “activation state.” In 2014, Butovsky et al. described a “homeostatic” microglial signature, comparing microglia with monocytic populations and other CNS cells (22). This signature includes genes such as *P2ry12, Fclrs, Tmem119, Hexb, Mertk, Cx3cr1, Csf1r*, etc. that have been used in numerous studies thereafter to identify microglial cells. This was a fundamental step in distinguishing resident microglia from other tissue-resident macrophages and infiltrates in disease context. This “homeostatic” signature was more recently revised and extended to developmental stages in addition to adulthood by Matcovitch-Natan et al. (23). In this study, single-cell RNA-seq helped associate the microglial signature identified at each different age to the potential functions these cells take on during life. They pinpointed three different temporal stages of development, each linked to a particular signature: early microglia associated with proliferation and differentiation, pre-microglia related to neuronal development, and adult microglia.

It has recently been suggested that microglial heterogeneity peaks early during development and then reaches a minimum in the homeostatic adult brain, only to regain diversity in old age (24). In addition, some microglial subtypes have been based on surface markers and sometimes function [discussed in Stratoulias et al. (25)]. This has been mostly achieved through systematic transcriptional investigation of microglia in different contexts. However, because every study is done with different techniques (microarrays, bulk RNA-seq, single-cell RNA-seq, etc.), on different kinds of samples (whole brain, sorted microglia based on different gating strategies, microdissected microglia, sorted nuclei, etc.), and in different animal models, there is a risk for confusion of data. We believe that there is a need for an overview—by looking at the big picture, common patterns can be identified between studies that might otherwise have been overlooked.

In this review, we summarize and interpret transcriptomic studies on microglia from development, homeostasis, and disease states to bring to light a subpopulation common to all these different states. We discuss the factors inducing this subpopulation and its functional importance in all of the studied conditions. Finally, we provide a core signature for this subset and propose to systematize and unify the naming of this microglial subpopulation to clarify the literature and avoid redundancy in future studies. We propose to use a name already used in numerous studies and that accounts for these cells' expression signature: CD11c+ microglia.

## CD11c+ Microglia History, Discovery, and Identification

For long, microglia have been considered simply as macrophages, due to the belief that all macrophages emerged from the bone marrow. Consensus that a subset of microglia expressed CD11c was therefore at first difficult to achieve. CD11c was widely accepted as a marker for dendritic cells (DCs), to the extent that some studies have used it as the sole identifier for DCs. Added to this was the constant difficulty of discriminating CNS-resident parenchymal microglia from blood-derived myeloid cells, with which they share many markers [reviewed in Amici et al. (26)]. Until recently, it was indeed not possible to reliably discriminate microglia, especially activated microglia, from blood-derived monocytic myeloid cells, using morphology or routine myeloid markers. Panels of differentially expressed genes that can be used to distinguish microglia including TMEM119 (27) and the homeostatic marker P2RY12 (22) were however recently identified and validated in both homeostatic and disease conditions (28).

To our knowledge, the first observation of microglia expressing CD11c was made in human multiple sclerosis (MS) tissue by immunohistochemical analysis (29). One, however, cannot be completely certain of the exclusive microglial nature of the cells identified in this study based on the markers used and our current knowledge of myeloid cell marker expression patterns. The first report to explicitly identify CD11c+ cells in the CNS as microglia came from Butovsky et al. in 2006 (30). They identified populations of CD11c+ cells in a mouse model for Alzheimer's disease (AD) as microglia, based on their location and co-expression of isolectin B4 and CD11b, although these cells showed a dendritic morphology. The major point of interest in that study was the observation that all MHC-II+ microglia that engulfed amyloid β in the brain of glatiramer acetate (GA)-vaccinated transgenic (Tg)-AD mice co-expressed CD11c. Also, relevant to our subsequent studies, these cells could be stained with an antibody specific for insulin-like growth factor 1 (IGF1).

A “gold standard” for microglial identification remains their relatively low level of expression of CD45 in flow cytometry analyses (31). In the course of study of glial responses in the dentate gyrus to axonal transection in the entorhinal cortex (the Perforant Path lesion model), we noted a subpopulation of CD45^low^ CD11b+ CD11c+ cells in flow-cytometry profiles of cells isolated from lesion-reactive hippocampus. Their functional significance and whether they derived intraparenchymally or by immigration from bone marrow were not determined (Babcock and Owens, unpublished). Exactly similar cells were then observed in cuprizone-demyelinated corpus callosum (32, 33). These were described to express slightly higher levels of CD45 than their CD11c− counterparts, while remaining within the CD45^low^ gate (33, 34). In addition, they did not express CCR2 characteristic for infiltrating leukocytes and expressed high levels of CX3CR1 supporting their microglial status (33). Further analysis showed that CD11c+ microglia were also induced in experimental autoimmune encephalomyelitis (EAE) (33–35) and a mouse model for neuromyelitis optica (NMO) (33), as well as during postnatal development (24, 35–37).

In older studies, ambiguity in assigning CD45 levels resulted in CD11b+ CD11c+ populations in CNS of mice with EAE or infected with *Toxoplasma gondii* being identified as DCs (38), although, with hindsight, consideration of bimodal CD45 profiles allows that at least some of them may have been microglia. The fact that CD11c+ microglia express slightly higher CD45 levels than resting microglia may have contributed to uncertainty, and claims that DCs derived from microglia (38, 39) may need re-evaluation.

Relative CD45 levels as detected by flow cytometry are not as useful for histological discrimination. Depending on the antibodies and staining protocols used, microglia may even not be detected as CD45+ cells, or else cannot be distinguished from other CD45^hi^ cells. Similarly, CD11c promoter-driven fluorescent reporter transgenic mice cannot discriminate between the many cell types that can express or upregulate CD11c without co-staining for lineage-specific markers. Identification of CD11c+ microglia in such mice relies on interpretation of sometimes fortuitous observations that include consideration of a cell's morphology and location. Using an EYFP-CD11c transgenic strain, Bulloch et al. identified a small fraction of CD11c+ microglia that were immunoreactive for Mac-1, IBA1, CD45, and F4/80 (40). The parenchymal juxtavascular IBA1+ CD11b+ GFP-CD11c+ cells described by Prodinger et al. in a CD11c-GFP reporter mouse likely included microglia, although in a non-diseased mouse, they would only account for around 2% of them (41). Flow-cytometric analysis confirmed CD45^low^ GFP-CD11c+ cells in the CNS of these mice (42). The fact that they were MHC II-negative likely reflects that they derived from non-diseased tissue, unlike the EAE-derived cells that we described (34). Typical microglia markers and their functions are listed in Table 1.

**Table 1:** Microglia markers and their function.

	**Marker**	**Main functions**	**References**
Common in microglia	CD45	Pan-leukocyte protein with tyrosine phosphatase activity Controls adhesion in macrophages	(43)
	CD11b	Integrin family member Pairs with CD18 to form CR3, a receptor for complement C3bi, mediating complement-coated particle uptake Plays a role in synaptic pruning	(44)
	CX3CR1	Fractalkine receptor Controls microglia activation Mediates microglia–neuron interaction Participates in chemotaxis	(45)
	IBA1	Calcium-binding protein Key molecule in membrane ruffling and phagocytosis	(46)
	TMEM119	Surface protein Unknown function in the CNS	(27)
	FCRLS	Scavenger receptor Unknown function in the CNS	(22)
Specific to CD11c+ microglia	CD11c	Integrin family member Pairs with CD18 to form CR4, a receptor for complement C3bi, mediating complement-coated particle uptake Regulates the activation and proliferation of leucocytes	(47)
	CLEC7A	Pattern recognition receptor Regulates autophagy, phagocytosis, and the respiratory burst	(48)
	SPP1	Secreted glycophosphoprotein Plays a role in in cellular motility, adhesion and survival	(49)

## CD11c+ Microglia in Homeostatic Conditions

### In Development

Even before microglia were formally identified, the presence of fat-laden cells had been reported and suggested to be a part of the normal developing CNS (50–52), and to participate in either cell death processes (53) or myelin formation (54–56). Early after the initial description of microglial cells, neuroanatomists began to track and map microglia in the CNS. Del Rio-Hortega was the first to describe “fountains of microglia” in the developing brain, having amoeboid morphology and being preferentially located in the white matter (57). Already in 1925, Penfield reported that what he describes as “neuroglia of mesodermal origin” “were variously considered to be normal and having to do with myelination or to indicate an abnormal inflammatory process” (58).

In the mid- to late 1970s, with del Rio-Hortega's “fountains of microglia” in mind, these cells were investigated again using light and electron microscopy. Most studies describe round, amoeboid, highly vacuolated cells with fat-containing granules, which are found in developing white matter, particularly along unmyelinated axonal tracts in the corpus callosum of rabbits (59), rats (60), mice (61), birds (62), fish (63), and humans (64), as opposed to more highly ramified cells present in the gray matter. In all these studies, amoeboid or ovoid-shaped microglia invade the white matter before disappearing when increasing numbers of ramified microglia colonize the gray matter (peaking around postanatal day (P) 5 and disappearing around P10 to P15 in rodents). Multiple studies support this finding and extrapolate their potential function, stating either that they have enhanced phagocytic abilities for the elimination of apoptotic material coming from normal developmental cell death or that they participate in myelination (59, 60, 65–68). This involvement in myelination was reinforced by a study by Pont-Lezica et al. showing that microglial alteration early in development leads to impaired corpus callosum fasciculation (11). Their phagocytic abilities along with their morphology provoked debates regarding their origin (68), their fate (66), and even their microglia status with some studies modifying the nomenclature by referring to them as “brain macrophages” rather than “amoeboid microglia” (67, 68).

With the new notion of microglial phenotypes emerging, these early amoeboid microglia were hypothesized to have higher “activation” levels before becoming “deactivated” in a controlled manner, as this was believed to be temporarily helpful to scavenge debris coming from developmental cellular death. To corroborate this hypothesis, Hristova et al. attempted the first phenotypic analysis of these cells, and reported expression of high levels of integrins alpha X (*Itgax*, CD11c), alpha 4 (*Itga4*), alpha 5 (*Itga5*), and beta 2 (*Itgb2*) in microglia from periventricular white matter in comparison to cortical microglia at P7 by staining quantification in IBA1+ cells (37). In addition, *in situ* hybridization clearly showed transient *Igf1* and colony-stimulating factor 1 (*Csf1*) mRNA expression within microglial cells in the corpus callosum and periventricular white matter until approximately two postnatal weeks (37). In this study, expression of *Igf1* and *Csf1* by microglia were hypothesized to play a protective role, preventing axonal damage for instance, which has since then been confirmed in a study by Ueno et al. (12).

This finding was reinforced by our own study showing that microglial cells expressing high levels of *Itgax* and *Igf1* are present in the white matter (cerebellum and corpus callosum) of developing mouse brains particularly between P3 and P5 where they make up almost 20% of all microglia and decrease in numbers already at P7 before being almost completely undetectable by P28 (35). Presence of *Igf1*-expressing microglia in these locations in P5 brains was further confirmed by *in situ* hybridization (69). We performed RNA-seq on these cells between P3 and P5 after FACS-sorting based on CD45^dim^ CD11b+ CD11c+ gating comparing them to their CD11c− counterparts. We identified a robust neurodevelopmental gene signature for developmental CD11c+ microglia, including factors involved in astrocyte and neuronal differentiation, tissue remodeling, and myelinogenesis accompanied by downregulation of immune function-related genes. Of note, *Itgax, Itga4, Csf1*, and *Igf1*, which were highlighted in the Hristova study, were also part of this signature. Importantly, we demonstrated that *Igf1* expression by CD11c+ microglia during development is crucial for primary myelination. Indeed, selective deletion of *Igf1* specifically from CD11c+ cells led to myelination defects in P21 brains (35). Interestingly, all neonatal microglia expressed neuroectodermal genes including *Nestin*.

A concomitant study by Hagemeyer et al. similarly identified amoeboid microglia in the developing white matter of the corpus callosum and cerebellum particularly between P1 and P8 before being almost undetectable by P14 (70). Interestingly, they used a Mac-3 staining to identify these cells, reminiscent of a study by Valentino and Jones who reported Mac-3 expression in “fountain microglia” in a footnote (68). They identified a signature akin to the one we found (38 genes in common out of 61 upregulated genes including *Itgax, Csf1*, and *Igf1*) by comparing “fountain microglia” from corpus callosum at P7 with cortical microglia at the same age by whole-genome microarray (70). Of note, the study underscores that many of the most upregulated genes were related to a primed or activated microglial phenotype and they confirmed CD11c expression in the “fountain of microglia” cells with a reporter mouse. In addition, by depleting all microglia during the critical period of the first postnatal week, they showed that the number of oligodendrocyte progenitor cells was reduced and a long-lasting effect on myelination was induced into adulthood (70), in line with our own results.

Two recent studies used single-cell RNA-seq to elucidate microglial heterogeneity during development (24, 36). The Barres lab study used deep single-cell RNA-seq on microglial cells sorted based on CD11b+ gating and CD45 levels from six different brain regions at E14.5, P7, and P60 (24). They found a cluster of cells they named “proliferative region-associated microglia” (PAM), mainly found at P7 in the white matter, that have an amoeboid morphology and phagocytose newly formed oligodendrocytes (24). In addition, they reported enhanced expression of *Igf1* and *Itgax* in this cluster compared to any other at P7 or other time points. These cells were observed as early as E17.5 in the embryonic brain, their numbers peaking around P7 and were almost absent from P14 brains (24). All these features fit with CD11c+ microglia from our study and the historical “fountain of microglia” cells.

The Stevens lab used high-throughput RNA-seq on microglial cells from the whole brain sorted based on a CD45^dim^ CD11b^hi^ CX3CR1^hi^ gating at E14.5, P4–5, P30, P100, and P540 and in injury contexts, prioritizing high numbers of cells over depth of sequencing (36). They identified a cluster of cells exclusive for the P4–5 time point, which have an amoeboid morphology, express phagocytosis-related genes, and are restricted to the corpus callosum and cerebellum, associating closely with axonal tracts, which they named “axon tract-associated microglia” (ATM) (36). Again, the features of this subset resembled closely the features of CD11c+ microglia and “fountain of microglia” cells described above. Interestingly, their study showed no evidence for a sex bias, the number of cells associated to this cluster being similar for neonatal female and male pups (36).

In addition, Anderson et al. (71) described gene signatures of retinal microglia in P7 mice, 60% of which were found to express CD11c. The microglial signature in the P7 retina fit the signature associated to developmental CD11c+ microglia as *Itgax, Lpl, Clec7a*, and *Igf1* were enriched in sorted CD11c^hi^ vs. CD11c^low^ cells at P7, whereas *P2ry12* and *Tmem119* were downregulated (71).

**Figure 1:**
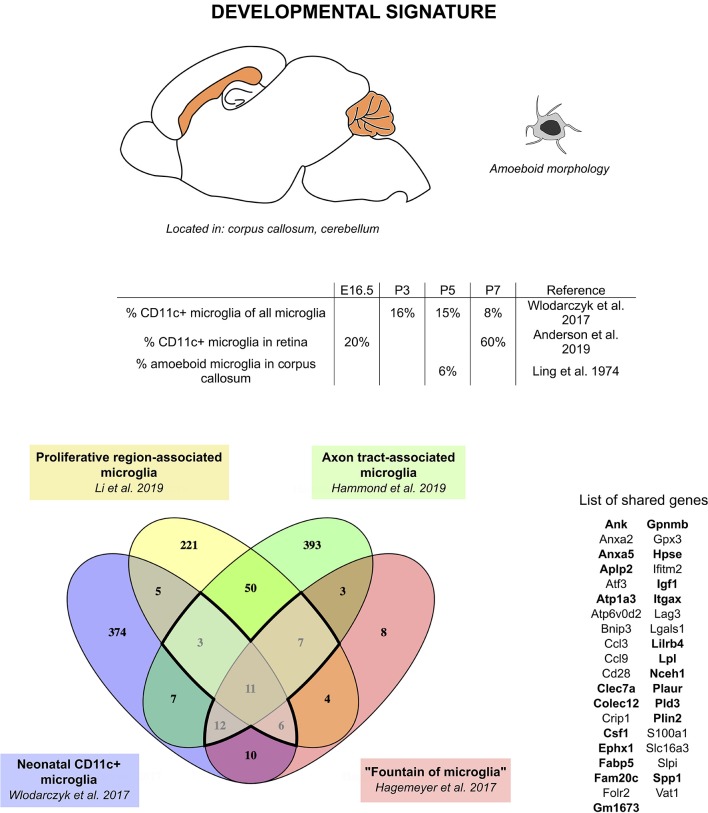
CD11c+ microglia signature in developmental stages. During development, CD11c+ microglia have an amoeboid morphology and localize close to white matter tracts, essentially in the corpus callous and cerebellum. They are present early during embryonic development and their numbers peak between P3 and P7. Comparison of genes upregulated in four studies (24, 35, 36, 70) reveals a common signature for developmental CD11c+ microglia of 39 genes upregulated in at least three of the studies (bold dark outline). Genes shared with the disease signature in Figure 2 are in bold. The Venn diagram was generated using the online tool Venny (72).

We therefore hypothesize that CD11c+ microglia, fountains of microglia, PAMs, and ATMs, although described in different studies by different methods under different names, actually represent the same population of cells. Comparison of the transcriptomic signature found in each of these studies leads to a core signature of 11 genes found in all four studies (*Gpnmb, Itgax, Spp1, Fam20c, Fabp5, Hpse, Igf1, Folr2, Csf1*, and *Anxa5*) and 28 additional genes found in at least three of these studies (*Atp6v0d2, Slpi, Cd28, Crip1, Lgals1, Anxa2, Vat1, Ifitm2, Gm1673, Plaur, S100a1, Colec12, Clec7a, Atf3, Atp1a3, Ephx1, Nceh1, Lpl, Pld3, Plin2, Aplp2, Ccl3, Bnip3, Ccl9, Gpx3, Slc16a3, Lag3*, and *Lilrb4*) (Figure 1). Interestingly, *Csf1*, one of the genes of the core signature, has been identified as one of the prominent genes characteristic of the pre-microglia homeostatic signature (23). These 39 genes constitute the “developmental signature” of the microglial population described in this section. Of note, homeostatic microglia markers, such as *Tmem119, P2ry12, Sall1, Tgfbr1, Fcrls*, and *Cx3cr1*, have been shown to be expressed by this subset, although in most reports at slightly lower levels than in adult microglia or other neonatal microglia (24, 35, 36, 70). Later in this review, we will refer to this population as “developmental CD11c+ microglia”. Features of this population include peak numbers between P3 and P7, amoeboid morphology, phagocytic abilities, and location in white matter (Figure 1). In addition, studies mentioned in this section clearly reveal a critical functional role of developmental CD11c+ microglia in the myelination process. Their presence in high numbers in the white matter makes them strategically placed in both space and time to take on that role. The aforementioned data support their involvement in phagocytosis of newly formed oligodendrocytes, probably linked to the proper establishment of primary myelination (24, 35, 36, 70). Two of the studies show the long-term importance of these cells on oligodendrocytes and myelination later in life (35, 70).

Although the number of common genes in the developmental signature might appear low, we would argue that this is probably due to discrepancies in the transcriptomic techniques used (microarray, bulk RNA-seq, high-throughput single-cell RNA-seq, deep single-cell RNA-seq), as well as the isolation techniques used (FACS-sorting based on various gatings, presence or absence of perfusion, whole brain dissection, or region microdissection) (see Table 2) [discussed in (76)]. However, similarities in the localization, colonization kinetics, morphology, and functional role leave little room for doubt regarding the uniqueness of the population described.

**Table 2:** Specification of studies used to establish the signatures.

	**References**	**Condition**	**Tissue**	**Isolation technique**	**Transcriptomic technique**
Development	(35)	P4–6	Whole brain	**FACS** CD45^dim^CD11b^+^CD11c^+^ *compared to* CD45^dim^CD11b^+^CD11c^−^	**RNA-seq** Illumina HiSeq 2500
	(70)	P7	Cortex *compared to* Corpus callosum	**FACS** CD45^dim^CD11b^+^Gr1^−^	**Microarray** Affymetrix Mouse Gene 2.0 ST Arrays
	(24)	P7	Cortex Cerebellum Hippocampus Striatum Olfactory bulb Choroid plexus	**FACS** CD45^+^CD11b^+^	**Single cell RNA-seq** Smart-seq 2 Illumina NextSeq
	(36)	P4–5	Whole brain	**FACS** CD45^dim^CD11b^+^CX3CR1^+^	**Single-cell RNA-seq** Chromium (10 × genomics) Illumina NextSeq 500
Diseases	(73)	APP/PS1 18 months	Cortex	**MACS** CD11c^+^ sorted fraction *compared to* CD11c^−^ eluted CD11b^+^ sorted	**Microarray** Agilent Technologies Mouse GE 4x44k V2 microarrays
	(74)	5XFAD 6 months	Whole brain	**FACS** CD45^+^	**MARS-seq** Illumina NextSeq 500
	(75)	APP/PS1 9 months	Whole brain	**FACS** FCRLS^+^CLEC7A^+^ *compared to* FCRLS^+^CLEC7A^−^	**RNA-seq** Illumina NextSeq 500
	(35)	Symptomatic EAE	Whole brain	**FACS** CD45^dim^CD11b^+^CD11c^+^ *compared to* CD45^dim^CD11b^+^CD11c^−^	**RNA-seq** Illumina HiSeq 2500

### In Adulthood

Recent studies have described the homeostatic adult brain as the state with lowest microglial heterogeneity (24). In addition, most high-throughput studies investigating adult microglia in steady state generally report very homogeneous populations in the homeostatic clusters, whether by mass cytometry (77) or single-cell RNA-seq (36), characterized by robust expression of classical microglial homeostatic markers.

However, in a CD11c-eYFP reporter mouse, YFP-expressing cells have been found throughout the brain and retina in adulthood. Although initially thought to be DCs (40), they have since then been shown to exhibit a phenotype resembling microglia (41, 78). Interestingly, a particular abundance of these cells is found in ventral areas of the brain, white matter tracts, and areas of adult neurogenesis (78). This is in line with a report that CLEC7A+ microglia are found in neurogenic niches in the adult mouse (24), showing that in the homeostatic adult brain, microglia with a phenotype similar to developmental CD11c+ microglia could remain in low number in selected areas. Consistent with this, a subset of microglia (also positive for TMEM119 and P2RY12) expressing higher levels of CD11c was found in the human subventricular zone and thalamus (79). In reporter mice, expression of CD11c has been shown to not always follow the expression of the YFP reporter and should therefore be taken cautiously (78). The existence of CD11c-expressing microglia has however been confirmed in the adult homeostatic brain (around 2% of total microglia) (33–35, 42, 80, 81). Similarly, a small population of cells from the choroid plexus of adult mice was shown to be transcriptionally distinct from other choroid plexus cells and border-associated brain macrophages. This population named “Kolmer's epiplexus cells” closely resembles microglial cells and was associated with enriched expression of *Spp1, Apoe*, and *Igf1* (82). Although *Itgax* was not among the significantly upregulated genes in this study, CD11c+ cells expressing low levels of CD45 have previously been described in the choroid plexus of adult mice (78).

### In Aging

Change in microglial gene expression and phenotype in steady-state aging has been studied extensively. Although reports agree on the changes in morphology and general phenotype of microglia toward dystrophic microglia (deramification, cytorrhexis, and fragmentation) in aging [reviewed in (83)], genomic studies have given discrepant results, with some arguing for shift toward neuroprotection (84) and others highlighting a “primed phenotype” with higher immune activation (85). That said, having a second look at datasets from various studies brings to light common highly expressed genes in aged microglia compared to young microglia: *Spp1, Clec7a, Igf1, Lpl, Axl, Apoe, Lgals3, Itgax, Cst7*, etc. are indeed found across several studies (84–86), although not all and not always in the same range of upregulation (87). In a later study, Holtman et al. related the “primed” microglial signature they found from two aging models (one physiological aging model and one accelerated aging model) to the study by Hickman et al. and found a high correlation between the datasets (88).

High-throughput single-cell methods are a good way to decipher complex populations with mixed subsets. A mass-cytometry study revealed that a specific subset of microglia emerges during aging that overexpresses surface CD11c and CD14, CLEC7A, and CD68 as compared to other microglia at the same age, although they downregulate CX3CR1 and MERTK (77, 89). CD11c expression of microglia in the white matter and caudal areas of the CNS of aged mice was also shown using immunohistochemistry (90). This study also reports expression of CLEC7A in white matter tracts of aged animals and reports numerous changes in white matter microglia associated with aging. Similarly, single-cell RNA-seq revealed that several populations of microglia that were present in younger age at very low numbers become increasingly prevalent with aging. One of these populations (referred to as OA2) is characterized by genes from the developmental signature and genes classically associated with neurodegeneration (*Spp1, Lpl, Lgals3, Lilrb4, Cst7, Apoe, Fam20c, Anxa5, Plaur, Aplp2*, etc.) among others (36). By showing the existence of a mix of different microglial subsets in the context of aging, this study helps us understand the seemingly discrepant results obtained by bulk RNA-seq performed on whole brain microglia during aging.

## Evidence for CD11c+ Microglia Signature in Repopulation Studies

Under homeostatic conditions, microglia are long-lived, self-renewing cells. Although some studies suggest that microglia persist throughout the life of an individual (91), others show that their turnover rate is quite fast, at around 1% per day in the mouse (92, 93) and 28% per year in the human (94). Regardless, their relatively long lifespan has been proposed to be crucial in microglial priming and ultimately contributing to neurodegeneration (91). Similarly, microglia have been found to be detrimental in some disease contexts [reviewed in Wolf et al. (95)], leading researchers to entertain the idea of transient microglial depletion as a therapeutic strategy (96, 97). Indeed, the depleted microglial niche gets repopulated within a couple of weeks post-depletion (98, 99). It is not yet resolved whether this repopulation occurs from peripheral cells or from a local microglial progenitor, and whether this progenitor is Nestin-positive. Such depletion strategies have had either beneficial or detrimental outcomes, depending on the pathology and the depletion method [reviewed in Han et al. (100)]. More recently, studies have characterized repopulating microglia, to assess whether and how they differ from the original microglia and whether these differences could account for the positive outcomes of microglial depletion strategies. Although morphological differences have been reported (101), most studies focused on gene expression analysis (98, 101–104). Two of the early studies advocated for repopulating microglia being functionally similar to resident control microglia (98, 101). However, closer examination and more recent studies, including single-cell RNA-seq, suggest that these cells differ transcriptionally (98, 102–104). Interestingly, Zhan et al. compared the repopulating microglial signature to the neonatal microglial signature (104), putting forward the idea that newly formed microglia resemble developmental microglia, before adopting a more mature phenotype. When comparing their transcriptomic data to the CD11c+ neonatal microglia signature we describe above, we found nine overlapping genes (*Atp6v0d2, Clec7a, Spp1, Lgals1, Gm1673, Gpnmb, Atp1a3, Itgax*, and *Ank*). Similarly, seven genes (*Atp6v0d2, Spp1, Igf1, Gpx3, Gpnmb, Ccl3*, and *Lpl*) overlapped with the repopulating microglial signature from Bruttger et al. (98), possibly indicating the presence of CD11c+-microglia-like cells in the repopulating clusters they described. This is reinforced by our study in which CD11c+ microglia could be found in repopulating microglia clusters after genetic microglial depletion (35). However, in contrast to Zhan et al. our analysis did not show neonatal-like, neurodevelopmental gene signature in repopulated microglia (35). The low extent of overlap between these studies and our newly defined neonatal CD11c+ microglia could be explained by heterogeneity of repopulating microglia, diluting the signal from CD11c+ microglia in bulk RNA-seq studies.

## CD11c+ Microglia in Disease States

Microglia activation is a common feature in many neurological disorders including inflammatory, demyelinating, and degenerative diseases, as well as glioma and injury. Although microglia activation may have deleterious consequences, it has also been shown in many instances to exert protective and regenerative effects. It is now becoming clear that there is an emergence of CD11c+ microglia population in pathological conditions. In this section, we will discuss the importance and the role of this cell subset in several neurological diseases.

### Alzheimer's Disease

For decades, it has been known that microglia localize around Aβ plaques, and engulf Aβ in AD, showing their importance in the disease. In recent years, interest in these cells has increased, largely due to a wave of transcriptomic and genome-wide association (GWAS) studies. In addition, a majority of AD risk genes are related to microglia, including triggering receptor induced on myeloid cells 2 (TREM2) [reviewed in McQuade and Blurton-Jones (105)]. Despite the enormous amount of data generated, no consensus has yet been reached on whether microglia are protective or detrimental in neurodegeneration. Some of the attempts to resolve this issue involved comparing transcriptomes of microglia sorted from healthy, aged, and diseased brains. The study by Holtman et al. cited above identified a microglial signature found not only in aging models but also in disease models including the APP/PS1 AD model and the SOD1 model for amyotrophic lateral sclerosis (ALS) (88). The common genes included *Itgax, Clec7a, Axl, Lgals3*, and *Apoe*, indicating the presence of a CD11c-expressing microglial population in these models. The gene module described in this study mostly contained genes related to phagocytosis and cell proliferation, with tissue protective elements (88). With a similar strategy, other studies demonstrated that microglia from aging brains and from amyloidosis (APP/PS1) and tauopathy (AAV-Tau P301L) shared a common gene signature including *Cst7, Itgax, Gpnmb, Clec7a, Lpl, Lgals3, Apoe*, and *Spp1* (86). Similar results were also obtained by Krasemann et al. in the APP/PS1 model. Such shared microglial characteristics led to the term “microglial neurodegenerative phenotype (MGnD) signature” (75). This is also in line with the presence of CD11c-expressing microglia in these models, with a phenotype similar to the one found in physiological aging.

The presence of CD11c+ microglia around Aβ plaques has been shown in several studies (30, 73, 74, 106, 107). A recent study by Kamphuis et al. extensively investigated the localization, proliferation status, and transcriptome of CD11c+ vs. CD11c− microglia in APP/PS1 mice (73). Importantly, this study also highlighted a steady increase in CD11c transcripts in brains of APP/PS1 and 3xTg-AD mice with aging as plaques appear, as well as in hippocampal samples from AD patients, although it declines in the later stages of the disease (73). The transcriptomic signature of CD11c+ microglia, when compared to their CD11c− counterparts, showed increased expression of *Gpnmb, Fabp5, Spp1, Igf1, Itgax, Gm1673, Cst7, Cox6a2, Apoe, Ch25h, Clec7a, Lilrb4, Csf1, Axl, Lpl, Sulf2, Egr2, Anxa5, Cd68, Timp2*, and *Ctsb* among others. Many of these genes are common with the developmental signature of CD11c+ microglia described above or with the signatures found in whole brain “primed” microglial signatures (73). These findings further support that the “primed” microglia phenotype described in many studies recapitulates the CD11c+ microglia signature diluted among CD11c− counterparts. The robustness of the signature is hardly surprising, considering that CD11c+ microglia make up for 23% of all Iba1+ cells in the aged APP/PS1 brain (73). Of note, strong upregulation of some CD11c+ microglia signature genes, including *Itgax, Clec7a*, and *Cst7*, was even detectable in whole tissue samples from cortex and hippocampus in AD models (73, 108).

High-throughput single-cell studies also contributed to our understanding of microglial populations in AD rodent models. The same study that identified CD11c and CD14 surface expression by mass cytometry on a microglia population emerging in aging also identified a similar population in APP/PS1 brains (77). Single-cell RNA-seq studies identified three microglial signatures in neurodegeneration models: the disease-associated microglia (DAM) signature (74), the late response microglia signature (109), and the activated response microglia (ARM) signature (80) that emerge in the 5xFAD, CK-p25, and APP^NL−G−F^ models for AD, respectively. All three studies described cell clusters showing nearly identical microglia populations, similar to the CD11c+ microglia signature observed in the Kamphuis study. Importantly, all of the DAM cells were CD11c+ (74) with highly overlapping gene signatures uncovered by bulk sequencing of sorted CD11c+ microglia (73). Microglia with characteristics from the ARM cluster are present in low numbers (ca. 2%) even in wild-type mice at young age, increasing as part of normal aging to reach up to about 12% of all microglia (80), consistent with observations discussed above of CD11c+ microglia in the steady state in adult and aging mice. ARM microglia are however most evident in APP^NL−G−F^ mice where they outnumber all other microglial clusters reaching 52% of all microglia at 21 months of age (80). This is in line with increases in CD11c+ microglia reported in other studies. Importantly, the signature observed in CD11c+/DAM/MGnD/ARM microglia is enriched for known AD risk genes (80). Of note, this transcriptomic signature is similar to that induced by retinal degeneration (110).

CD11c+ microglia have been demonstrated to be beneficial for and to correlate with increased Aβ uptake and induction of IGF1-mediated neurogenesis in an animal model of AD (30). In addition, abundance of *Igf1*-expressing microglia around Aβ plaques was recently confirmed by *in situ* hybridization in an AD model (69). Functional analyses led to discrepant results suggesting either protective, immunosuppressive function as well as enhanced capacity for uptake and lysosomal degradation of Aβ (73), or pathogenicity via possible contribution to local arginine deprivation and subsequent neurodegeneration (111). Butovsky's group also proposed a detrimental role for these cells due to ameliorated Aβ deposition in 4-month-old TREM2-deficient mice that lack CD11c+ microglia (75). However, the role of TREM2 is not clear, since other data show either protective or detrimental roles for this protein depending on the age of the animals (75, 112–114). Nonetheless, all these studies demonstrate lack of microglial proliferation and clustering around plaques in TREM2-deficient animals, thus allowing for more dispersed Aβ localization in AD models (75, 112–116). This can be detrimental due to Aβ spreading that is not limited by microglia clusters, ultimately leading to severe axonal dystrophy (114). Moreover, it has been demonstrated that in TREM2-deficient animals older than 8 months, the Aβ burden is enhanced as compared to 4-month-old animals, suggesting that TREM2 signaling is necessary for limiting advanced stage pathology (117). Thus, CD11c+ microglia may actually be beneficial and protective in later stages of the disease as proposed by Keren-Shaul et al. (74). Human data further support this hypothesis since loss-of-function mutations in TREM2 have been identified as a strong risk factor for the development of AD and other neurodegenerative diseases [reviewed in McQuade and Blurton-Jones and Ulland and Colonna (105, 118)].

Collectively, CD11c+ microglia (also referred to as primed microglia, late response microglia, DAM, ARM, or MGnD) are a well-defined population of cells that show adaptation predominantly for phagocytic clearance of apoptotic/necrotic neurons and limiting Aβ spreading. Given that AD risk genes are enriched in this population (80), mutations in such genes may have an impact on the ability of CD11c+ microglia to cope with Aβ plaque burden, either promoting or limiting AD pathology.

### Amyotrophic Lateral Sclerosis

ALS is a disease affecting motor neurons leading to their degeneration. Microglial contribution to the disease has been established since a robust microglial activation has been found in both patient and transgenic mouse tissue (119, 120). In addition, many risk factors for the disease have been shown to be expressed by microglia in the CNS, reinforcing the idea of an involvement of these cells in the disease (121). Microglial activation in the disease arises from accumulation of misfolded protein, and, similarly to observations made in other disease contexts, microglia have been reported to play a beneficial role in the pre-symptomatic phase of the disease before shifting to detrimental roles in the advanced disease state (122). However, microglial depletion in the context of ALS has not been found to increase survival (123), leading to the idea that both functions might be concomitant, constantly counteracting each other. Interestingly, a study from 2013 analyzed the transcriptome of microglia sorted from mice carrying an ALS-associated mutation and found a particular signature for these cells at the end stage of the disease compared to microglia from healthy brains (124). Once again, among the top regulated genes were genes related to Huntington's disease, AD, and Parkinson's disease (*Mapt, Psen2, Apoe*, etc.). The signature found in this study includes both factors reported to be beneficial in the context of ALS (*Igf1, Grn, Trem2, Tyrobp*, etc.), and factors known to be detrimental (*Mmp12, Optn, Cybb*, etc.), as well as some like *Spp1, Gpnmb*, and *Itgax* recurrently found in neurodegenerative diseases. Microglia were also found to upregulate surface CD11c. Microglia from SOD1 mice were also found to fit the abovementioned MGnD signature, in addition to expressing *Clec7a* levels increasingly during disease progression (75).

### Stroke, Ischemia, and Injury

Neuron degeneration and nerve injury have been linked to microglia in various models for traumatic brain injury (TBI) (125), spinal cord injury (SCI) (126), nerve injury (93), and ischemic stroke (127). Much like in inflammation models, microglial contribution in all of these models is still rather unclear and they may play a double role considering their association with both beneficial and detrimental effects. Studying microglia in context of inflammation can get quite complicated due to massive infiltration of peripheral immune cells, notably monocytes and macrophages, occurring subsequently to TBI (128), SCI (129), and stroke (127, 130, 131). In a study comparing the transcriptomics of microglia and macrophages after ischemia in rats, it was reported that microglia played a detrimental role and macrophages played a beneficial role with regard to recovery, based on their expression of classical inflammation markers (132). Investigation of the genes enriched in microglia three days after middle cerebral artery occlusion compared to sham controls, however, revealed *Spp1, Gpnmb, Lgals3, Fabp5*, and *Axl* among others, fitting with the potential presence of CD11c+ microglia-like cells in this context, diluted among other microglia. Consistent with this, *Ccl2* mRNA was found to be increased in microglia and macrophages at this time point (132), an aspect that has been associated with the emergence of CD11c+ microglia (81). Another study, conducted in a model of phototrombic stroke on whole tissue, actually showed upregulation of *Gpnmb, Itgax*, and *Clec7a* in a cluster associated with early response (133), which the authors related to the DAM phenotype (74). In a study of facial nucleus axotomy, the authors also related the observed microglial phenotype (134) to the DAM phenotype, as well as to a phenotype found in the Ck-p25 model (109): 72 genes were regulated in common between all three studies representing almost 75% of all genes upregulated in the facial nucleus axotomy model. Interestingly, in an SCI transcriptomic study, a profile of microglia reminiscent of the CD11c+ phenotype was identified (with upregulation of *Gpnmb, Spp1, Lpl, Apoe, Igf1, Lgals3*, and *Itgax* among others) and persisted in a full transection model, whereas it contracted concomitantly to recovery in a hemisection model (135), indicative of the transitory nature of this subset. Conversely, in TBI, the microglial signature was further from the CD11c+ microglia signature, although *Itgax* was among the upregulated genes 14 and 60 days post-injury, possibly indicating once again a dilution of the signature in all microglia (136). In addition, considering the difficulty associated with gating out macrophages from microglia in a context of extensive infiltration, macrophage contamination of the sorted samples cannot be excluded in these studies, potentially complicating interpretation of the observed transcriptomes.

### Multiple Sclerosis

MS is an inflammatory, demyelinating disease of the CNS that can be modeled by EAE or toxin-induced demyelinating models. Recent advancement in our understanding of the disease points toward important roles for microglia in the pathomechanism. Although the evidence supporting their implication in initiation and facilitation of the disease is strong (95), there is a growing body of evidence for their protective functions including involvement in remyelination (137).

We have identified CD11c+ microglia during EAE accounting for around 10% of total microglia in whole CNS (33, 34). Of note, this subset is even more abundant in the spinal cord at the peak of the diseases reaching up to 60% of total microglia (Wlodarczyk, unpublished). The emergence of the CD11c+ microglia is a dynamic process starting at the onset, reaching a maximum at the peak and contracting in the chronic phase of EAE (77, 138). These cells are localized in the demyelinated spinal cord lesions (33). CD11c+ microglia from EAE again showed upregulation of similar genes as in neurodegenerative models including *Itgax, Gpnmb, Spp1*, etc. (35). A similar signature was confirmed by Krasemann et al. (75). In addition, deep analysis of genes that were upregulated in CD11c+ microglia population pointed to their involvement in immune responses (35).

A key aspect of neuroinflammation in EAE is the recruitment and reactivation of encephalitogenic T cells to express their effector functions. Many cell types are implicated in this process, including blood-derived DCs and monocytes/macrophages but also parenchymal microglia (139). In EAE, CD11c+ microglia express MHCI, MHCII, and costimulatory molecules CD80/CD86 (34, 140), which is in line with recent high-throughput mass-cytometry reports (77, 138). We have provided evidence that CD11c+ microglia are able to induce similar proliferative response of encephalitogenic CD4+ T cells as blood-derived professional antigen-presenting cells (32, 34). Interestingly, in contrast to CD11c+ blood-derived cells and CD11c− microglia, CD11c+ microglia completely lacked mRNA expression for IL-23 (34) that is known to induce GM-CSF-producing CD4+ T cells, critical for EAE pathology (141). This indicates that although CD11c+ microglia alone might contribute to T cell expansion, they are unlikely to induce pathogenic T cell responses. Importantly, a subsequent study showed that they were a major source of message for myelinogenic IGF1, suggesting that they might exert protective roles in EAE (33). This is supported by our recent study showing that stimulation of CSF1R with its ligands during symptomatic EAE significantly reduced demyelination and ameliorated disease progression most likely through induction of CD11c+ microglia (81). Moreover, decreasing CD11c+ microglia by blocking of TREM2 signaling (as discussed below) led to increased severity of EAE and exacerbated demyelinating lesions in the spinal cord (142), further supporting protective roles of CD11c+ microglia.

Microglia are known to contribute to remyelination by creating an environment supporting OPC recruitment and differentiation by phagocytosing myelin debris, secreting growth factors and modulating extracellular matrix [reviewed in Lloyd and Miron (137)]. Circumstantial evidence for remyelinating properties of CD11c+ microglia includes our first demonstration of the expansion of these cells in cuprizone-demyelinated corpus callosum (32). A microarray study by Olah et al. identified a pro-remyelinating microglial signature that includes several genes reminiscent of the CD11c+ microglia characteristics described above (*Itgax Igf1, Clec7a, Apoe, Spp1*) (143). Moreover, CD11c immunoreactive microglia were present in remyelinating corpus callosum (32). A similar microglial signature was later confirmed in both demyelination and remyelination phases (144). Conversely, microglia expressing the CD11c+ microglia signature including *Apoe, Axl, Igf1, Lyz2, Itgax*, and *Gpnmb* were identified by single-cell transcriptomics in both de- and remyelinated lesions (145). Recently, cuprizone-mediated demyelination was shown to be alleviated in mice lacking microglial SIRPα that have increased numbers of CD11c+ microglia, pointing to their protective role (89). In line with the induction of CD11c+ microglia (81), stimulation of CSF1R ameliorated cuprizone-induced demyelination (146).

Another line of evidence comes from the influence of TREM2 deficiency, which leads to absence of CD11c+ microglia in adult mice (74, 75), on remyelination after cuprizone demyelination. The data indicate that TREM2 deficiency had no impact on the initial demyelination, but affected subsequent remyelination when the cuprizone treatment was prolonged, most likely by impairing myelin removal as well as myelin regeneration, which further supports a protective role for CD11c+ microglia in this paradigm (144, 147). Additionally, it was reported that microglial necroptosis in circumstances of lysophosphatidylcholine demyelination leads to repopulation by pro-regenerative CD11c+ microglia, as blocking of this mechanism prevented remyelination (148). Of note, demyelination induced by mouse hepatitis virus also led to enrichment of CD11c+ microglial gene signature in the spinal cord (149).

Taken together, association of CD11c+ microglia to white matter (89) as well as their role in primary myelination strongly support their importance in induction and facilitation of remyelination. This opens the possibility for induction of innate repair programs in diseased CNS via promotion of the emergence of CD11c+ microglia.

### Glioma

Very early studies identified microglial cells close to gliomas to resemble the amoeboid form described during development and to take on phagocytic functions (58). More recent studies have shown that parenchymal microglia are attracted to the tumor in glioma-affected brains, representing up to 30% of the tumor mass (150). Microglia associated to the tumor have been termed glioma-associated microglia/macrophages (GAM). These cells initially exhibit beneficial anti-tumor abilities but have been found to be hijacked by the tumor to exert tumor-promoting functions [reviewed in Li and Graeber (151)]. A study from 2015 identified a signature for GAMs, and emphasized their high expression of SPP1 and GPNMB (152). They compared this signature to classical macrophage activation markers (M1/M2) and concluded a lack of overlap between the GAM signature and these classical phenotypes. Of note, the signature also includes genes such as *Itgax, Fabp5*, and *Clec7a* among others recurrently found in disease signatures (152).

### Microglial Disease Signature

**Figure 2:**
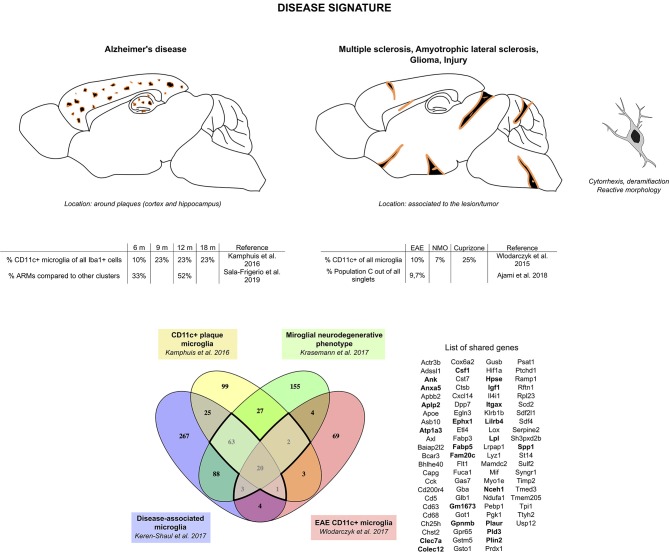
CD11c+ microglia signature in disease states. In diseased CNS, CD11c+ microglia adopt an amoeboid, reactive morphology. In AD, they are found surrounding Aβ plaques. Similarly, in MS and ALS models and in injury, they are found around and in the lesions. In glioma, they are found mixed with tumor cells. CD11c+ microglia numbers in diseased CNS vary considerably, ranging from 10 to 50% of all microglia. Comparison of genes upregulated in four studies (35, 73–75) reveals a common signature for CD11c+ disease microglia of 89 genes upregulated in at least three of the studies (bold dark outline). Genes shared with the developmental signature in Figure 1 are in bold. Raw data for the Krasemann study were obtained using the Gene Expression Omnibus Database and the differential gene expression analysis was performed using the DEBrowser package in R (153). The Venn diagram was generated using the online tool Venny (72).

Considering the similarities observed in gene expression from the different studies aforementioned, we compared the transcriptomic signatures obtained in studies comparing specifically microglia sorted based on a typical marker for this specific subset of microglia or from single-cell RNA-seq (three of the AD studies and one EAE study, Figure 2). We found a core disease signature for microglia consisting of 89 genes shared between all four studies (Figure 2). *Itgax* being once again a part of this signature and with clarity in mind, we will refer to this signature as the “CD11c+ microglia disease signature” henceforth. Once again, the microglial nature of this subset is supported by expression, although slightly lower than in homeostatic microglia, of *Tmem119, Cx3cr1, P2ry12, Sall1*, and *Tgfbr1* among other homeostatic genes (35, 73–75).

## CD11c+ Microglia Signature

Over the years, advancements in technology have allowed the scientific community to investigate cells and cell populations in increasingly detailed ways, particularly at the molecular level. This investigation has been done using a multiplicity of different conditions and models, leading to increasing amounts of data generated. Although invaluable, this work has also led to redundancy in the microglial profiles that were identified (154).

Our investigation led us to define two particularly strong signatures for CD11c+ microglia in development (Figure 1) and in disease (Figure 2). Interestingly, Li et al. (24) as well as Anderson et al. (71) related the developmental microglia signature observed in their studies to the DAM microglial signature. These similarities prompted us to compare the signatures we identified from the literature.

**Figure 3:**
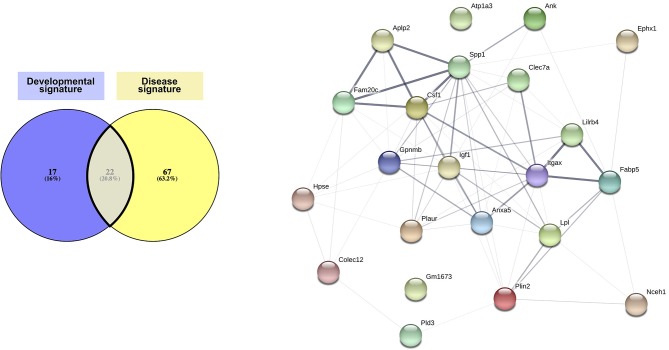
Core CD11c+ microglia signature. Considering similarities between the transcriptomic signatures and functions in the CD11c+ microglia subset in development and in disease, we compared both signatures to obtain a core of genes upregulated in this subset across all conditions. We observe overlap of 20% of the genes between both signatures, corresponding to 22 shared genes. Upon interrogating the STRING database (Szklarczyk D, Gable AL, Lyon D, Junge A, Wyder S, Huerta-Cepas J, Simonovic M, Doncheva NT, Morris JH, Bork P, Jensen LJ, von Mering C. STRING v11: protein-protein association networks with increased coverage, supporting functional discovery in genome-wide experimental datasets. *Nucleic Acids Res*. 2019 Nov; 47:D607–613.), we observed that the network formed by the proteins corresponding to the genes in the core signature had significantly more interactions than expected from a similar set of random proteins, indicating that these proteins related to the genes in the core signature are at least partially biologically connected. The thickness of the edges linking the different genes is proportional to the strength of the evidence linking the two proteins. The Venn diagram was generated using the online tool Venny (72).

Comparison of the developmental signature and the disease signature resulted in defining of a “core” signature common to CD11c+ microglia across all contexts, which consists of 22 genes: *Ank, Anxa5, Aplp2, Atp1a3, Clec7a, Colec12, Csf1, Ephx1, Fabp5, Fam20c, Gm1673, Gpnmb, Hpse, Igf1, Itgax, Lilrb4, Lpl, Nceh1, Plaur, Pld3, Plin2*, and *Spp1* (Figure 3 and Supplementary Table 1). Interestingly, the protein network linked to these genes had significantly more links than what can be expected, indicating at least a partial biological connection between these genes (Figure 3). Further investigation of the physiological function of the proteins related to the genes present in the core signature revealed their involvement in lipid metabolism, cell migration and proliferation, and, to a lesser extent, immune function (Supplementary Table 1). As expected, all of these proteins had been associated with various brain diseases (Supplementary Table 1). Of note, many of these proteins assume similar function or have been found to interact directly or indirectly with each other (Supplementary Table 1). Further investigation of these genes and proteins in link with one another would most likely unveil interesting mechanisms underlying CD11c+ microglia function.

Although described previously as different microglial subsets, we argue that the robust core signature we have identified can be found for this subset across all these different stages. We suggest that the differences in this subset observed between conditions reflect methodological discrepancies (Table 2) or microenvironment-linked context-specific changes and the subset's own phenotypic plasticity in coping with these variations, rather than fundamental differences in cell lineage.

## Emergence of CD11c+ Microglia

**Figure 4:**
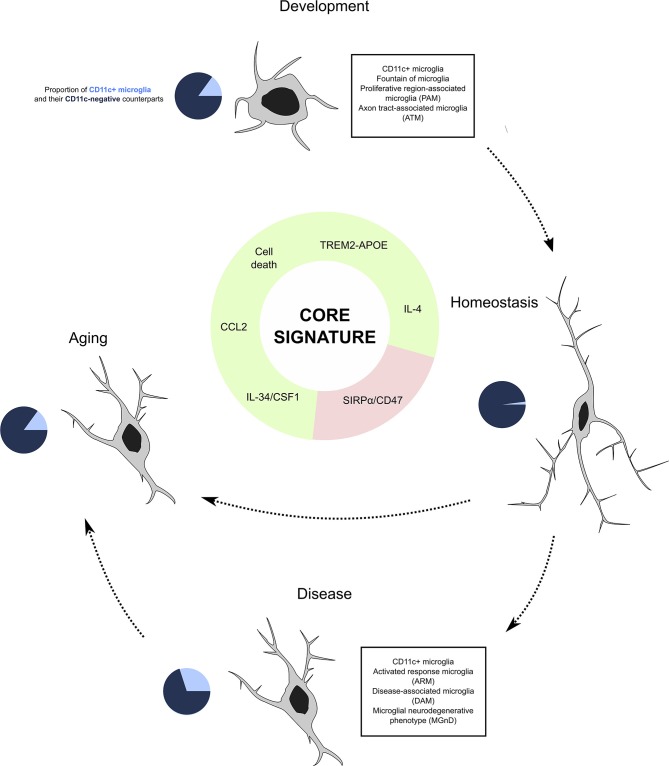
CD11c+ microglia as a subset of microglia present through life and across conditions. Our investigation leads us to believe that CD11c+ microglia represent a subset of microglia characterized by a robust signature of 22 genes expressed by this subset at any age and in various disease states. Emergence of this subset is induced by various factors including signaling through the TREM2–APOE pathway, cell death, IL-4 signaling, and cytokine signaling through CSF1R inducing CCL2, and is inhibited by CD47/SIRPα signaling. In physiological conditions, CD11c+ microglia account for around 15% of all microglia, before contracting to 2% in adulthood and being re-induced by aging at levels similar to development. In disease states, their numbers oscillate between 10 and 50%. We argue that despite the numerous names given to this subset across conditions, it is unique and should be referred to as “CD11c+ microglia”.

The dynamics of CD11c+ microglia seem tightly spatio-temporally regulated. They first emerge during the first postnatal week, peaking at P5 and gradually decreasing as animals age, being barely detectable in the healthy adult CNS (33–35, 42, 80, 81) to increase again in aging or disease (33, 73, 81, 85, 89). Importantly, none of the studies that have investigated induction of inflammation by means of lipopolysaccharide, poly(I:C), or other immune challenges could recapitulate the robust CD11c+ signature found in steady state and disease and injury contexts (86, 88, 90, 124, 155, 156). Below, we present factors that participate in controlling the induction of this population (Figure 4).

### Activation of the TREM2–APOE Pathway

One candidate that has been extensively studied with regard to CD11c+ microglia is the TREM2 pathway. TREM2-deficient animals were shown to downregulate the CD11c+ microglia signature in cuprizone-induced demyelination (144) and in an AD model (113). In addition, in the study from the Amit lab, TREM2 deficiency in an AD mouse model led to an arrest of microglia in an intermediate state between the homeostatic state and the CD11c+ microglia stage. Barely any microglia in these mice exhibited the CD11c+ microglial signature (74). This suggests that CD11c+ microglia induction is a two-step process, where the first step, to leave the homeostatic state, is TREM2-independent and the second step, to reach the complete CD11c+ microglia phenotype, is TREM2-dependent. These observations were confirmed by Krasemann et al. in another TREM2-deficient AD model (75). Similarly, APOE-deficient mice exhibit lower numbers of CD11c+ microglia in AD, ALS, and MS mouse models (75, 80). This is suggestive of a positive feedback loop, as this population itself strongly upregulates APOE (75). Surprisingly, the Barres lab showed that induction of CD11c+ microglia during postnatal development in contrast to adulthood is TREM2–APOE-independent (24). A similar TREM2 independence of CD11c+ microglia induction was shown in the developing retina (71).

### Cell Death

Krasemann et al. highlighted phagocytosis of apoptotic neurons and monocytes as a trigger for the induction of the CD11c+ microglia phenotype (75). Of note, induction of this phenotype was not observed upon microglia exposure to *Escherichia coli*, zymosan particles (75), or microparticles (Marczynska et al., unpublished), suggesting that induction of CD11c+ microglia is a tightly controlled reaction to local cell damage or apoptosis, rather than to phagocytosis itself. Interestingly, microglial necroptosis in demyelination models leads to brain repopulation with CD11c+ microglia from nestin+ resident microglia (148). Similarly, nestin+ microglia colonizing the brain after microglia ablation expressed surface CD11c (98). The gene expression in repopulating microglia highly overlapped with the CD11c+ microglia signature. We showed that genetic or toxin-induced ablation of neonatal CD11c+ cells led to their instant repopulation (35). Whether the observed concomitant decrease of CD11c− microglia (35) reflects induction of CD11c+ phenotype in CD11c− cells by phagocytosis of dying microglia has not been determined. Interestingly, a dramatic decrease in CD11c+ microglia was observed in the postnatal retina of mice deficient in *Bax*, a pro-apoptotic gene that is essential for developmental death of neurons (71). This emphasizes that apoptotic cells are a strong and common inducer of CD11c+ microglia regardless of age and condition. This is also in line with several studies where developmental cell death has been linked to microglial entry in the developing CNS (61). In addition, retinal CD11c+ microglia were resistant to depletion induced by either CSF1R deficiency or blocking, contrary to their CD11c− counterparts. In line with this, our own data showed that despite using several depletion regimens, CD11c+ microglia could not be depleted from postnatal brain as they were immediately repopulated (35).

### Cytokines

We have shown that both populations of adult microglia (CD11c+ and CD11c−) express equal levels of CSF1R (33). Importantly, stimulation of this receptor by its ligands, interleukin (IL)-34 and CSF1, induced a significant increase in CD11c+ microglia numbers, with faster kinetics for IL-34 (81). Moreover, such stimulation induced CCL2 in the brain, and we showed that overexpression of CCL2 leads to a dramatic expansion of CD11c+ microglia in a CCR2-independent manner (81).

Butovsky et al., on the other hand, showed that another cytokine, IL-4, can induce CD11c+ expression on Aβ pretreated microglia (30, 157). Moreover, they demonstrated that GA vaccination leads to an increase of CD11c+ microglia surrounding Aβ plaques and suggested that this was induced by T-cell-derived IL-4 (30).

### Inhibition of SIRPα/CD47 Signaling

Recently, the emergence of CD11c+ microglia in the adult brain has been shown to be homeostatically controlled by SIRPα/CD47 interaction. Genetic ablation of SIRPα in microglia or global lack of CD47 equally resulted in increased numbers of CD11c+ microglia, suggesting that microglial SIRPα suppresses CD11c expression in the same cells (89).

## Conclusion

Here, we have demonstrated that the subpopulation of microglia described in many recent studies (and named PAM, ATM, fountain of microglia, DAM, ARM, MGnD, and late response microglia) indeed reflects the characteristics of CD11c+ microglia, originally identified over a decade ago. Thus, we believe that a unification of the nomenclature by referring to the microglial subset expressing the described signature, from development to old age, as CD11c+ microglia is a necessary step to progress our understanding of microglia biology. This subset emerges in development before contracting during adulthood but is triggered to re-emerge in aging as well as in the context of disease or tissue injury (Figure 4). The summary of the data that mentioned microglia showing the aforementioned signature strongly points to the importance of CD11c+ microglia in primary myelination during CNS development as well as their protective, remyelinative, and regenerative capacities in CNS pathology. This opens new perspectives for therapeutic targeting of microglia in neurological conditions.

## Author Contributions

AW and AB-B designed the manuscript. AB-B analyzed the transcriptomic data and prepared the figures. AB-B, AW, and TO wrote and approved the manuscript.

### Conflict of Interest

The authors declare that the research was conducted in the absence of any commercial or financial relationships that could be construed as a potential conflict of interest.

## References

[1] del Río-Hortega P El “tercer elemento” de los centros nerviosos. I. La microglía en estado normal. II. Intervención de la microglía en los procesos patológicos (células en bastoncito y cuerpos gránulo-adiposos). III. Naturaleza probable de la microglía. Bol Soc Esp Biol. 69–120.

[2] Ginhoux F Greter M Leboeuf M Nandi S See P Gokhan S. Fate mapping analysis reveals that adult microglia derive from primitive macrophages. Science. (2010) 330:841–5. 10.1126/science.1194637 20966214 PMC3719181

[3] Alliot F Godin I Pessac B. Microglia derive from progenitors, originating from the yolk sac, and which proliferate in the brain. Dev Brain Res. (1999) 117:145–52. 10.1016/S0165-3806(99)00113-3 10567732

[4] Monier A Adle-Biassette H Delezoide A-L Evrard P Gressens P Verney C. Entry and distribution of microglial cells in human embryonic and fetal cerebral cortex. J Neuropathol Exp Neurol. (2007) 66:372–82. 10.1097/nen.0b013e3180517b46 17483694

[5] Menassa DA Gomez-Nicola D. Microglial dynamics during human brain development. Front Immunol. (2018) 9:1014. 10.3389/fimmu.2018.01014 29881376 PMC5976733

[6] Miyamoto A Wake H Ishikawa AW Eto K Shibata K Murakoshi H. Microglia contact induces synapse formation in developing somatosensory cortex. Nat Commun. (2016) 7:1–2. 10.1038/ncomms12540 27558646 PMC5007295

[7] Parkhurst CN Yang G Ninan I Savas JN Yates JR Lafaille JJ. Microglia promote learning-dependent synapse formation through brain-derived neurotrophic factor. Cell. (2013) 155:1596–609. 10.1016/j.cell.2013.11.030 24360280 PMC4033691

[8] Paolicelli RC Bolasco G Pagani F Maggi L Scianni M Panzanelli P. Synaptic pruning by microglia is necessary for normal brain development. Science. (2011) 333:1456–8. 10.1126/science.1202529 21778362

[9] Weinhard L di Bartolomei G Bolasco G Machado P Schieber NL Neniskyte U. Microglia remodel synapses by presynaptic trogocytosis and spine head filopodia induction. Nat Commun. (2018) 9:1228. 10.1038/s41467-018-03566-5 29581545 PMC5964317

[10] Squarzoni P Oller G Hoeffel G Pont-Lezica L Rostaing P Low D. Microglia modulate wiring of the embryonic forebrain. Cell Rep. (2014) 8:1271–79. 10.1016/j.celrep.2014.07.042 25159150

[11] Pont-Lezica L Beumer W Colasse S Drexhage H Versnel M Bessis A. Microglia shape corpus callosum axon tract fasciculation: functional impact of prenatal inflammation. Eur J Neurosci. (2014) 39:1551–7. 10.1111/ejn.12508 24593277

[12] Ueno M Fujita Y Tanaka T Nakamura Y Kikuta J Ishii M. Layer V cortical neurons require microglial support for survival during postnatal development. Nat Neurosci. (2013) 16:543–51. 10.1038/nn.3358 23525041

[13] Arnò B Grassivaro F Rossi C Bergamaschi A Castiglioni V Furlan R. Neural progenitor cells orchestrate microglia migration and positioning into the developing cortex. Nat Commun. (2014) 5:1–13. 10.1038/ncomms6611 25425146

[14] Cunningham CL Martínez-Cerdeño V Noctor SC. Microglia regulate the number of neural precursor cells in the developing cerebral cortex. J Neurosci. (2013) 33:4216–33. 10.1523/JNEUROSCI.3441-12.2013 23467340 PMC3711552

[15] Davalos D Grutzendler J Yang G Kim JV Zuo Y Jung S. ATP mediates rapid microglial response to local brain injury *in vivo*. Nat Neurosci. (2005) 8:752–8. 10.1038/nn1472 15895084

[16] Nimmerjahn A Kirchhoff F Helmchen F. Resting microglial cells are highly dynamic surveillants of brain parenchyma *in vivo*. Science. (2005) 308:1314–8. 10.1126/science.1110647 15831717

[17] Liu YU Ying Y Li Y Eyo UB Chen T Zheng J. Neuronal network activity controls microglial process surveillance in awake mice via norepinephrine signaling. Nat Neurosci. (2019) 22:1771–81. 10.1038/s41593-019-0511-3 31636449 PMC6858573

[18] Torres L Danver J Ji K Miyauchi JT Chen D Anderson ME. Dynamic microglial modulation of spatial learning and social behavior. Brain Behav Immun. (2016) 55:6–16. 10.1016/j.bbi.2015.09.001 26348580 PMC4779430

[19] Greenhalgh AD David S Bennett FC. Immune cell regulation of glia during CNS injury and disease. Nat Rev Neurosci. (2020) 21:139–52. 10.1038/s41583-020-0263-9 32042145

[20] Ransohoff RM Perry VH. Microglial physiology: unique stimuli, specialized responses. Annu Rev Immunol. (2009) 27:119–45. 10.1146/annurev.immunol.021908.132528 19302036

[21] Ransohoff RM. A polarizing question: do M1 and M2 microglia exist? Nat Neurosci. (2016) 19:987–91. 10.1038/nn.4338 27459405

[22] Butovsky O Jedrychowski MP Moore CS Cialic R Lanser AJ Gabriely G. Identification of a unique TGF-β-dependent molecular and functional signature in microglia. Nat Neurosci. (2014) 17:131–43. 10.1038/nn.3599 24316888 PMC4066672

[23] Matcovitch-Natan O Winter DR Giladi A Aguilar SV Spinrad A Sarrazin S. Microglia development follows a stepwise program to regulate brain homeostasis. Science. (2016) 353:aad8670. 10.1126/science.aad8670 27338705

[24] Li Q Cheng Z Zhou L Darmanis S Neff NF Okamoto J. Developmental heterogeneity of microglia and brain myeloid cells revealed by deep single-cell RNA sequencing. Neuron. (2019) 101:207–23.e10. 10.1016/j.neuron.2018.12.006 30606613 PMC6336504

[25] Stratoulias V Venero JL Tremblay M-È Joseph B. Microglial subtypes: diversity within the microglial community. EMBO J. (2019) 38:e101997. 10.15252/embj.2019101997 31373067 PMC6717890

[26] Amici SA Dong J Guerau-de-Arellano M. Molecular mechanisms modulating the phenotype of macrophages and microglia. Front Immunol. (2017) 8:1520. 10.3389/fimmu.2017.01520 29176977 PMC5686097

[27] Bennett ML Bennett FC Liddelow SA Ajami B Zamanian JL Fernhoff NB. New tools for studying microglia in the mouse and human CNS. Proc Natl Acad Sci USA. (2016) 113:E1738–46. 10.1073/pnas.1525528113 26884166 PMC4812770

[28] Haage V Semtner M Vidal RO Hernandez DP Pong WW Chen Z. Comprehensive gene expression meta-analysis identifies signature genes that distinguish microglia from peripheral monocytes/macrophages in health and glioma. Acta Neuropathol Commun. (2019) 7:1–18. 10.1186/s40478-019-0665-y 30764877 PMC6376799

[29] Ulvestad E Williams K MøRk S Antel J Nyland H. Phenotypic differences between human monocytes/macrophages and microglial cells studied *in situ* and *in vitro*. J Neuropathol Exp Neurol. (1994) 53:492–501. 10.1097/00005072-199409000-00008 8083690

[30] Butovsky O Koronyo-Hamaoui M Kunis G Ophir E Landa G Cohen H. Glatiramer acetate fights against Alzheimer's disease by inducing dendritic-like microglia expressing insulin-like growth factor 1. Proc Natl Acad Sci USA. (2006) 103:11784–9. 10.1073/pnas.0604681103 16864778 PMC1544247

[31] Ford AL Goodsall AL Hickey WF Sedgwick JD. Normal adult ramified microglia separated from other central nervous system macrophages by flow cytometric sorting. Phenotypic differences defined and direct *ex vivo* antigen presentation to myelin basic protein-reactive CD4+ T cells compared. J Immunol. (1995) 154:4309–21. 7722289

[32] Remington LT Babcock AA Zehntner SP Owens T. Microglial recruitment, activation, and proliferation in response to primary demyelination. Am J Pathol. (2007) 170:1713–24. 10.2353/ajpath.2007.060783 17456776 PMC1854965

[33] Wlodarczyk A Cédile O Jensen KN Jasson A Mony JT Khorooshi R. Pathologic and protective roles for microglial subsets and bone marrow- and blood-derived myeloid cells in central nervous system inflammation. Front Immunol. (2015) 6:463. 10.3389/fimmu.2015.00463 26441968 PMC4562247

[34] Wlodarczyk A Løbner M Cédile O Owens T. Comparison of microglia and infiltrating CD11c+ cells as antigen presenting cells for T cell proliferation and cytokine response. J Neuroinflammation. (2014) 11:57. 10.1186/1742-2094-11-57 24666681 PMC3987647

[35] Wlodarczyk A Holtman IR Krueger M Yogev N Bruttger J Khorooshi R. A novel microglial subset plays a key role in myelinogenesis in developing brain. EMBO J. (2017) 36:3292–308. 10.15252/embj.201696056 28963396 PMC5686552

[36] Hammond TR Dufort C Dissing-Olesen L Giera S Young A Wysoker A. Single-cell RNA sequencing of microglia throughout the mouse lifespan and in the injured brain reveals complex cell-state changes. Immunity. (2019) 50:253–71.e6. 10.1016/j.immuni.2018.11.004 30471926 PMC6655561

[37] Hristova M Cuthill D Zbarsky V Acosta-Saltos A Wallace A Blight K. Activation and deactivation of periventricular white matter phagocytes during postnatal mouse development. Glia. (2010) 58:11–28. 10.1002/glia.20896 19544386

[38] Fischer H-G Reichmann G. Brain dendritic cells and macrophages/microglia in central nervous system inflammation. J Immunol. (2001) 166:2717–26. 10.4049/jimmunol.166.4.2717 11160337

[39] Reichmann G Schroeter M Jander S Fischer H-G. Dendritic cells and dendritic-like microglia in focal cortical ischemia of the mouse brain. J Neuroimmunol. (2002) 129:125–32. 10.1016/S0165-5728(02)00184-4 12161028

[40] Bulloch K Miller MM Gal-Toth J Milner TA Gottfried-Blackmore A Waters EM. CD11c/EYFP transgene illuminates a discrete network of dendritic cells within the embryonic, neonatal, adult, and injured mouse brain. J Comp Neurol. (2008) 508:687–710. 10.1002/cne.21668 18386786

[41] Prodinger C Bunse J Krüger M Schiefenhövel F Brandt C Laman JD. CD11c-expressing cells reside in the juxtavascular parenchyma and extend processes into the glia limitans of the mouse nervous system. Acta Neuropathol. (2011) 121:445–58. 10.1007/s00401-010-0774-y 21076838

[42] Immig K Gericke M Menzel F Merz F Krueger M Schiefenhövel F. CD11c-positive cells from brain, spleen, lung, and liver exhibit site-specific immune phenotypes and plastically adapt to new environments. Glia. (2015) 63:611–25. 10.1002/glia.22771 25471735

[43] Saunders AE Johnson P. Modulation of immune cell signalling by the leukocyte common tyrosine phosphatase, CD45. Cell Signal. (2010) 22:339–48. 10.1016/j.cellsig.2009.10.003 19861160

[44] Schafer DP Lehrman EK Kautzman AG Koyama R Mardinly AR Yamasaki R. Microglia sculpt postnatal neural circuits in an activity and complement-dependent manner. Neuron. (2012) 74:691–705. 10.1016/j.neuron.2012.03.026 22632727 PMC3528177

[45] Arnoux I Audinat E. Fractalkine signaling and microglia functions in the developing brain. Neural Plast. (2015) 2015:1–8. 10.1155/2015/689404 26347402 PMC4539507

[46] Ohsawa K Imai Y Sasaki Y Kohsaka S. Microglia/macrophage-specific protein Iba1 binds to fimbrin and enhances its actin-bundling activity. J Neurochem. (2004) 88:844–56. 10.1046/j.1471-4159.2003.02213.x 14756805

[47] Erdei A Lukácsi S Mácsik-Valent B Nagy-Baló Z Kurucz I Bajtay Z. Non-identical twins: different faces of CR3 and CR4 in myeloid and lymphoid cells of mice and men. Semin Cell Dev Biol. (2019) 85:110–21. 10.1016/j.semcdb.2017.11.025 29174917

[48] Brown GD. Dectin-1: a signalling non-TLR pattern-recognition receptor. Nat Rev Immunol. (2006) 6:33–43. 10.1038/nri1745 16341139

[49] Icer MA Gezmen-Karadag M. The multiple functions and mechanisms of osteopontin. Clin Biochem. (2018) 59:17–24. 10.1016/j.clinbiochem.2018.07.003 30003880

[50] Hayem G Delahaye A Études Sur Les Diverses Formes D'encéphalite: (Anatomie Et Physiologie Pathologiques) Paris: A. Delahaye (1868).

[51] Jastrowitz M Studien über die encephalitis und myelitis des ersten kindesalters. Arch Für Psychiatr Nervenkrankh. (1870) 2:389–414. 10.1007/BF02046645

[52] Merzbacher L Untersuchungen über die Morphologie und Biologie der Abraümzellen im Zentralnervesystem. Fischer Verlag (1909).

[53] Parrot J-M-J Étude de la stéatose interstitielle diffuse de l'encéphale chez le nouveau-né. Arch Physiol Norm Pathol. (1868) 1:530–550; 622–642; 706–715.

[54] Boll FC Die histologie und histogenese der nervösen centralorgane. Arch Psychiatr. (1874) 4:1–138. 10.1007/BF02346085

[55] Eichhorst H Über die entwicklung des menschlichen rückenmarks und seiner Formelemente. Virchows Arch. (1875) 64:425–75. 10.1007/BF01991422

[56] Fleichsig P Die Leitungsbahnen im Gehirn und Rückenmark des Menschen auf Grund Entwicklungsgeschichtlicher Untersuchungen Dargestellt. Leipzig: Engelmann (1876).

[57] Penfield W Cytology & Cellular Pathology of the Nervous System. New york, NY: P.B. Hoeber, Inc (1932).

[58] Penfield W. Microglia and the process of phagocytosis in gliomas. Am J Pathol. (1925) 1:77–90. 19969634 PMC1931672

[59] Stensaas LJ Reichert WH. Round and amoeboid microglial cells in the neonatal rabbit brain. Z Für Zellforsch Mikrosk Anat. (1971) 119:147–63. 10.1007/BF00324517 5569842

[60] Ling EA Tan CK. Amoeboid microglial cells in the corpus callosum of neonatal rats. Arch Histol Jpn Nihon Soshikigaku Kiroku. (1974) 36:265–80. 10.1679/aohc1950.36.265 4857968

[61] Perry VH Hume DA Gordon S. Immunohistochemical localization of macrophages and microglia in the adult and developing mouse brain. Neuroscience. (1985) 15:313–26. 10.1016/0306-4522(85)90215-5 3895031

[62] Cuadros MA Martin C Coltey P Almendros A Navascués J. First appearance, distribution, and origin of macrophages in the early development of the avian central nervous system. J Comp Neurol. (1993) 330:113–29. 10.1002/cne.903300110 8468399

[63] Herbomel P Thisse B Thisse C. Zebrafish early macrophages colonize cephalic mesenchyme and developing brain, retina, and epidermis through a M-CSF receptor-dependent invasive process. Dev Biol. (2001) 238:274–88. 10.1006/dbio.2001.0393 11784010

[64] Rezaie P Male D. Colonisation of the developing human brain and spinal cord by microglia: a review. Microsc Res Tech. (1999) 45:359–82. 10.1002/(SICI)1097-0029(19990615)45:6<359::AID-JEMT4>3.0.CO;2-D 10402264

[65] Imamoto K Leblond CP. Radioautographic investigation of gliogenesis in the corpus callosum of young rats II. Origin of microglial cells. J Comp Neurol. (1978) 180:139–63. 10.1002/cne.901800109 649786

[66] Ling EA. Transformation of monocytes into amoeboid microglia in the corpus callosum of postnatal rats, as shown by labelling monocytes by carbon particles. J Anat. (1979) 128:847–58. 489472 PMC1232886

[67] Milligan CE Cunningham TJ Levitt P. Differential immunochemical markers reveal the normal distribution of brain macrophages and microglia in the developing rat brain. J Comp Neurol. (1991) 314:125–35. 10.1002/cne.903140112 1797868

[68] Valentino KL Jones EG Morphological and immunocytochemical identification of macrophages in the developing corpus callosum. Anat Embryol. (1981) 163:157–72. 10.1007/BF00320673

[69] Myhre CL Thygesen C Villadsen B Vollerup J Ilkjær L Krohn KT. Microglia express insulin-like growth factor-1 in the hippocampus of aged APPswe/PS1ΔE9 transgenic mice. Front Cell Neurosci. (2019) 13:308. 10.3389/fncel.2019.00308 31417357 PMC6682662

[70] Hagemeyer N Hanft K-M Akriditou M-A Unger N Park ES Stanley ER. Microglia contribute to normal myelinogenesis and to oligodendrocyte progenitor maintenance during adulthood. Acta Neuropathol. (2017) 134:441–58. 10.1007/s00401-017-1747-1 28685323 PMC5951721

[71] Anderson SR Roberts JM Zhang J Steele MR Romero CO Bosco A. Developmental apoptosis promotes a disease-related gene signature and independence from CSF1R signaling in retinal microglia. Cell Rep. (2019) 27:2002–13.e5. 10.1016/j.celrep.2019.04.062 31091440 PMC6544177

[72] Oliveros JC Venny. An Interactive Tool For Comparing Lists With Venn's Diagrams. (2007). Available online at: https://bioinfogp.cnb.csic.es/tools/venny/index.html (accessed October 15, 2019).

[73] Kamphuis W Kooijman L Schetters S Orre M Hol EM. Transcriptional profiling of CD11c-positive microglia accumulating around amyloid plaques in a mouse model for Alzheimer's disease. Biochim Biophys Acta. (2016) 1862:1847–60. 10.1016/j.bbadis.2016.07.007 27425031

[74] Keren-Shaul H Spinrad A Weiner A Matcovitch-Natan O Dvir-Szternfeld R Ulland TK. A unique microglia type associated with restricting development of Alzheimer's disease. Cell. (2017) 169:1276–90.e17. 10.1016/j.cell.2017.05.018 28602351

[75] Krasemann S Madore C Cialic R Baufeld C Calcagno N El Fatimy R. The TREM2-APOE pathway drives the transcriptional phenotype of dysfunctional microglia in neurodegenerative diseases. Immunity. (2017) 47:566–81.e9. 10.1016/j.immuni.2017.08.008 28930663 PMC5719893

[76] Hirbec HE Noristani HN Perrin FE Microglia responses in acute and chronic neurological diseases: what microglia-specific transcriptomic studies taught (and did not teach) us. Front Aging Neurosci. (2017) 9:227 10.3389/fnagi.2017.00227 28785215 PMC5519576

[77] Mrdjen D Pavlovic A Hartmann FJ Schreiner B Utz SG Leung BP High-dimensional single-cell mapping of central nervous system immune cells reveals distinct myeloid subsets in health, aging, and disease. Immunity. (2018) 48:380–95.e6. 10.1016/j.immuni.2018.01.011 29426702

[78] Dando SJ Golborne CN Chinnery HR Ruitenberg MJ McMenamin PG A case of mistaken identity: CD11c-eYFP+ cells in the normal mouse brain parenchyma and neural retina display the phenotype of microglia, not dendritic cells. Glia. (2016) 64:1331–49. 10.1002/glia.23005 27189804

[79] Böttcher C Schlickeiser S Sneeboer MAM Kunkel D Knop A Paza E. Human microglia regional heterogeneity and phenotypes determined by multiplexed single-cell mass cytometry. Nat Neurosci. (2019) 22:78. 10.1038/s41593-018-0290-2 30559476

[80] Sala Frigerio C Wolfs L Fattorelli N Thrupp N Voytyuk I Schmidt I. The major risk factors for alzheimer's disease: age, sex, and genes modulate the microglia response to Aβ plaques. Cell Rep. (2019) 27:1293–306.e6. 10.1016/j.celrep.2019.03.099 31018141 PMC7340153

[81] Wlodarczyk A Benmamar-Badel A Cédile O Jensen KN Kramer I Elsborg NB. CSF1R stimulation promotes increased neuroprotection by CD11c+ microglia in EAE. Front Cell Neurosci. (2019) 12:523. 10.3389/fncel.2018.00523 30687013 PMC6335250

[82] Hove HV Martens L Scheyltjens I Vlaminck KD Antunes ARP Prijck SD. A single-cell atlas of mouse brain macrophages reveals unique transcriptional identities shaped by ontogeny and tissue environment. Nat Neurosci. (2019) 22:1021. 10.1038/s41593-019-0393-4 31061494

[83] Verkhratsky A Zorec R Rodriguez-Arellano JJ Parpura V Neuroglia in ageing. In: Verkhratsky A Ho MS Zorec R Parpura V, editors. Neuroglia in Neurodegenerative Diseases Advances in Experimental Medicine Biology. Singapore: Springer (2017) p. 181–97. 10.1007/978-981-13-9913-8_8 PMC7188603 31583589

[84] Hickman SE Kingery ND Ohsumi TK Borowsky ML Wang L Means TK. The microglial sensome revealed by direct RNA sequencing. Nat Neurosci. (2013) 16:1896–905. 10.1038/nn.3554 24162652 PMC3840123

[85] Raj D Yin Z Breur M Doorduin J Holtman IR Olah M. Increased white matter inflammation in aging- and alzheimer's disease brain. Front Mol Neurosci. (2017) 10:206. 10.3389/fnmol.2017.00206 28713239 PMC5492660

[86] Kang SS Ebbert MTW Baker KE Cook C Wang X Sens JP. Microglial translational profiling reveals a convergent APOE pathway from aging, amyloid, and tau. J Exp Med. (2018) 215:2235–45. 10.1084/jem.20180653 30082275 PMC6122978

[87] Orre M Kamphuis W Osborn LM Melief J Kooijman L Huitinga I. Acute isolation and transcriptome characterization of cortical astrocytes and microglia from young and aged mice. Neurobiol Aging. (2014) 35:1–14. 10.1016/j.neurobiolaging.2013.07.008 23954174

[88] Holtman IR Raj DD Miller JA Schaafsma W Yin Z Brouwer N. Induction of a common microglia gene expression signature by aging and neurodegenerative conditions: a co-expression meta-analysis. Acta Neuropathol Commun. (2015) 3:31. 10.1186/s40478-015-0203-5 26001565 PMC4489356

[89] Sato-Hashimoto M Nozu T Toriba R Horikoshi A Akaike M Kawamoto K. Microglial SIRPα regulates the emergence of CD11c+ microglia and demyelination damage in white matter. eLife. (2019) 8:e42025. 10.7554/eLife.42025 30910011 PMC6435324

[90] Hart AD Wyttenbach A Hugh Perry V Teeling JL. Age related changes in microglial phenotype vary between CNS regions: grey versus white matter differences. Brain Behav Immun. (2012) 26:754–65. 10.1016/j.bbi.2011.11.006 22155499 PMC3381227

[91] Füger P Hefendehl JK Veeraraghavalu K Wendeln A-C Schlosser C Obermüller U. Microglia turnover with aging and in an Alzheimer's model via long-term *in vivo* single-cell imaging. Nat Neurosci. (2017) 20:1371–6. 10.1038/nn.4631 28846081

[92] Askew K Li K Olmos-Alonso A Garcia-Moreno F Liang Y Richardson P. Coupled proliferation and apoptosis maintain the rapid turnover of microglia in the adult brain. Cell Rep. (2017) 18:391–405. 10.1016/j.celrep.2016.12.041 28076784 PMC5263237

[93] Tay TL Mai D Dautzenberg J Fernández-Klett F Lin G Sagar. A new fate mapping system reveals context-dependent random or clonal expansion of microglia. Nat Neurosci. (2017) 20:793–803. 10.1038/nn.4547 28414331

[94] Réu P Khosravi A Bernard S Mold JE Salehpour M Alkass K. The lifespan and turnover of microglia in the human brain. Cell Rep. (2017) 20:779–84. 10.1016/j.celrep.2017.07.004 28746864 PMC5540680

[95] Wolf SA Boddeke HWGM Kettenmann H. Microglia in physiology and disease. Annu Rev Physiol. (2017) 79:619–43. 10.1146/annurev-physiol-022516-034406 27959620

[96] Feng X Valdearcos M Uchida Y Lutrin D Maze M Koliwad SK. Microglia mediate postoperative hippocampal inflammation and cognitive decline in mice. JCI Insight. (2017) 2:e91229. 10.1172/jci.insight.91229 28405620 PMC5374063

[97] Rice RA Pham J Lee RJ Najafi AR West BL Green KN. Microglial repopulation resolves inflammation and promotes brain recovery after injury. Glia. (2017) 65:931–44. 10.1002/glia.23135 28251674 PMC5395311

[98] Bruttger J Karram K Wörtge S Regen T Marini F Hoppmann N. Genetic cell ablation reveals clusters of local self-renewing microglia in the mammalian central nervous system. Immunity. (2015) 43:92–106. 10.1016/j.immuni.2015.06.012 26163371

[99] Varvel NH Grathwohl SA Baumann F Liebig C Bosch A Brawek B. Microglial repopulation model reveals a robust homeostatic process for replacing CNS myeloid cells. Proc Natl Acad Sci USA. (2012) 109:18150–55. 10.1073/pnas.1210150109 23071306 PMC3497743

[100] Han J Zhu K Zhang X-M Harris RA. Enforced microglial depletion and repopulation as a promising strategy for the treatment of neurological disorders. Glia. (2019) 67:217–31. 10.1002/glia.23529 30378163 PMC6635749

[101] Elmore MRP Lee RJ West BL Green KN. Characterizing newly repopulated microglia in the adult mouse: impacts on animal behavior, cell morphology, and neuroinflammation. PLoS ONE. (2015) 10:e0122912. 10.1371/journal.pone.0122912 25849463 PMC4388515

[102] Huang Y Xu Z Xiong S Sun F Qin G Hu G. Repopulated microglia are solely derived from the proliferation of residual microglia after acute depletion. Nat Neurosci. (2018) 21:530–40. 10.1038/s41593-018-0090-8 29472620

[103] Lund H Pieber M Parsa R Han J Grommisch D Ewing E. Competitive repopulation of an empty microglial niche yields functionally distinct subsets of microglia-like cells. Nat Commun. (2018) 9:1–13. 10.1038/s41467-018-07295-7 30451869 PMC6242869

[104] Zhan L Krabbe G Du F Jones I Reichert MC Telpoukhovskaia M. Proximal recolonization by self-renewing microglia re-establishes microglial homeostasis in the adult mouse brain. PLoS Biol. (2019) 17:e3000134. 10.1371/journal.pbio.3000134 30735499 PMC6383943

[105] McQuade A Blurton-Jones M. Microglia in alzheimer's disease: exploring how genetics and phenotype influence risk. J Mol Biol. (2019) 431:1805–17. 10.1016/j.jmb.2019.01.045 30738892 PMC6475606

[106] Akiyama H McGeer PL. Brain microglia constitutively express β-2 integrins. J Neuroimmunol. (1990) 30:81–93. 10.1016/0165-5728(90)90055-R 1977769

[107] Manczak M Mao P Nakamura K Bebbington C Park B Reddy PH. Neutralization of granulocyte macrophage colony-stimulating factor decreases amyloid beta 1-42 and suppresses microglial activity in a transgenic mouse model of Alzheimer's disease. Hum Mol Genet. (2009) 18:3876–93. 10.1093/hmg/ddp331 19617638 PMC2748895

[108] Landel V Baranger K Virard I Loriod B Khrestchatisky M Rivera S. Temporal gene profiling of the 5XFAD transgenic mouse model highlights the importance of microglial activation in Alzheimer's disease. Mol Neurodegener. (2014) 9:33. 10.1186/1750-1326-9-33 25213090 PMC4237952

[109] Mathys H Adaikkan C Gao F Young JZ Manet E Hemberg M. Temporal tracking of microglia activation in neurodegeneration at single-cell resolution. Cell Rep. (2017) 21:366–80. 10.1016/j.celrep.2017.09.039 29020624 PMC5642107

[110] O'Koren EG Yu C Klingeborn M Wong AYW Prigge CL Mathew R. Microglial function is distinct in different anatomical locations during retinal homeostasis and degeneration. Immunity. (2019) 50:723–37.e7. 10.1016/j.immuni.2019.02.007 30850344 PMC6592635

[111] Kan MJ Lee JE Wilson JG Everhart AL Brown CM Hoofnagle AN. Arginine deprivation and immune suppression in a mouse model of Alzheimer's disease. J Neurosci. (2015) 35:5969–82. 10.1523/JNEUROSCI.4668-14.2015 25878270 PMC4397598

[112] Jay TR Miller CM Cheng PJ Graham LC Bemiller S Broihier ML. TREM2 deficiency eliminates TREM2+ inflammatory macrophages and ameliorates pathology in Alzheimer's disease mouse models. J Exp Med. (2015) 212:287–95. 10.1084/jem.20142322 25732305 PMC4354365

[113] Wang Y Cella M Mallinson K Ulrich JD Young KL Robinette ML. TREM2 lipid sensing sustains the microglial response in an Alzheimer's disease model. Cell. (2015) 160:1061–71. 10.1016/j.cell.2015.01.049 25728668 PMC4477963

[114] Yuan P Condello C Keene CD Wang Y Bird TD Paul SM. TREM2 haplodeficiency in mice and humans impairs the microglia barrier function leading to decreased amyloid compaction and severe axonal dystrophy. Neuron. (2016) 90:724–39. 10.1016/j.neuron.2016.05.003 27196974 PMC4898967

[115] Mazaheri F Snaidero N Kleinberger G Madore C Daria A Werner G. TREM2 deficiency impairs chemotaxis and microglial responses to neuronal injury. EMBO Rep. (2017) 18:1186–98. 10.15252/embr.201743922 28483841 PMC5494532

[116] Wang Y Ulland TK Ulrich JD Song W Tzaferis JA Hole JT. TREM2-mediated early microglial response limits diffusion and toxicity of amyloid plaques. J Exp Med. (2016) 213:667–75. 10.1084/jem.20151948 27091843 PMC4854736

[117] Jay TR Hirsch AM Broihier ML Miller CM Neilson LE Ransohoff RM. Disease progression-dependent effects of TREM2 deficiency in a mouse model of Alzheimer's disease. J Neurosci. (2017) 37:637–47. 10.1523/JNEUROSCI.2110-16.2016 28100745 PMC5242410

[118] Ulland TK Colonna M. TREM2 — a key player in microglial biology and Alzheimer disease. Nat Rev Neurol. (2018) 14:667–75. 10.1038/s41582-018-0072-1 30266932

[119] Hall ED Oostveen JA Gurney ME. Relationship of microglial and astrocytic activation to disease onset and progression in a transgenic model of familial ALS. Glia. (1998) 23:249–56. 9633809 10.1002/(sici)1098-1136(199807)23:3<249::aid-glia7>3.0.co;2-#

[120] McGeer PL McGeer EG. Inflammatory processes in amyotrophic lateral sclerosis. Muscle Nerve. (2002) 26:459–70. 10.1002/mus.10191 12362410

[121] Haukedal H Freude K. Implications of microglia in amyotrophic lateral sclerosis and frontotemporal dementia. J Mol Biol. (2019) 431:1818–29. 10.1016/j.jmb.2019.02.004 30763568

[122] Henkel JS Beers DR Zhao W Appel SH. Microglia in ALS: the good, the bad, and the resting. J Neuroimmune Pharmacol. (2009) 4:389–98. 10.1007/s11481-009-9171-5 19731042

[123] Gowing G Philips T Wijmeersch BV Audet J-N Dewil M Bosch LVD Ablation of proliferating microglia does not affect motor neuron degeneration in amyotrophic lateral sclerosis caused by mutant superoxide dismutase. J Neurosci. (2008) 28:10234–44. 10.1523/JNEUROSCI.3494-08.2008 18842883 PMC6671032

[124] Chiu IM Morimoto ETA Goodarzi H Liao JT O'Keeffe S Phatnani HP. A neurodegeneration-specific gene-expression signature of acutely isolated microglia from an amyotrophic lateral sclerosis mouse model. Cell Rep. (2013) 4:385–401. 10.1016/j.celrep.2013.06.018 23850290 PMC4272581

[125] Jassam YN Izzy S Whalen M McGavern DB El Khoury J. Neuroimmunology of Traumatic Brain Injury: Time for a Paradigm Shift. Neuron. (2017) 95:1246–65. 10.1016/j.neuron.2017.07.010 28910616 PMC5678753

[126] David S Kroner A. Repertoire of microglial and macrophage responses after spinal cord injury. Nat Rev Neurosci. (2011) 12:388–99. 10.1038/nrn3053 21673720

[127] Denes A Thornton P Rothwell NJ Allan SM. Inflammation and brain injury: acute cerebral ischaemia, peripheral and central inflammation. Brain Behav Immun. (2010) 24:708–23. 10.1016/j.bbi.2009.09.010 19770034

[128] Simon DW McGeachy MJ Bayir H Clark RSB Loane DJ Kochanek PM The far-reaching scope of neuroinflammation after traumatic brain injury. Nat Rev Neurol. (2017) 13:171–91. 10.1038/nrneurol.2017.13 28186177 PMC5675525

[129] Shechter R Miller O Yovel G Rosenzweig N London A Ruckh J. Recruitment of beneficial M2 macrophages to injured spinal cord is orchestrated by remote brain choroid plexus. Immunity. (2013) 38:555–69. 10.1016/j.immuni.2013.02.012 23477737 PMC4115271

[130] Miró-Mur F Pérez-de-Puig I Ferrer-Ferrer M Urra X Justicia C Chamorro A. Immature monocytes recruited to the ischemic mouse brain differentiate into macrophages with features of alternative activation. Brain Behav Immun. (2016) 53:18–33. 10.1016/j.bbi.2015.08.010 26275369

[131] Wattananit S Tornero D Graubardt N Memanishvili T Monni E Tatarishvili J. Monocyte-derived macrophages contribute to spontaneous long-term functional recovery after stroke in mice. J Neurosci. (2016) 36:4182–95. 10.1523/JNEUROSCI.4317-15.2016 27076418 PMC6601783

[132] Rajan WD Wojtas B Gielniewski B Gieryng A Zawadzka M Kaminska B. Dissecting functional phenotypes of microglia and macrophages in the rat brain after transient cerebral ischemia. Glia. (2018) 67:232–45. 10.1002/glia.23536 30485549

[133] Kaiser J Maibach M Salpeter I Hagenbuch N Souza VBC de Robinson MD. The spinal transcriptome after cortical stroke: in search of molecular factors regulating spontaneous recovery in the spinal cord. J Neurosci. (2019) 39:4714–26. 10.1523/JNEUROSCI.2571-18.2019 30962276 PMC6561692

[134] Tay TL Sagar Dautzenberg J Grün D Prinz M. Unique microglia recovery population revealed by single-cell RNAseq following neurodegeneration. Acta Neuropathol Commun. (2018) 6:87. 10.1186/s40478-018-0584-3 30185219 PMC6123921

[135] Noristani HN Gerber YN Sabourin J-C Le Corre M Lonjon N Mestre-Frances N. RNA-seq analysis of microglia reveals time-dependent activation of specific genetic programs following spinal cord injury. Front Mol Neurosci. (2017) 10:90. 10.3389/fnmol.2017.00090 28420963 PMC5376598

[136] Izzy S Liu Q Fang Z Lule S Wu L Chung JY. Time-dependent changes in microglia transcriptional networks following traumatic brain injury. Front Cell Neurosci. (2019) 13:307. 10.3389/fncel.2019.00307 31440141 PMC6694299

[137] Lloyd AF Miron VE. The pro-remyelination properties of microglia in the central nervous system. Nat Rev Neurol. (2019) 15:447–58. 10.1038/s41582-019-0184-2 31256193

[138] Ajami B Samusik N Wieghofer P Ho PP Crotti A Bjornson Z. Single-cell mass cytometry reveals distinct populations of brain myeloid cells in mouse neuroinflammation and neurodegeneration models. Nat Neurosci. (2018) 21:541–51. 10.1038/s41593-018-0100-x 29507414 PMC8629134

[139] Almolda B Gonzalez B Castellano B. Antigen presentation in EAE: role of microglia, macrophages and dendritic cells. Front Biosci. (2011) 16:1157–71. 10.2741/3781 21196224

[140] Lewis ND Hill JD Juchem KW Stefanopoulos DE Modis LK. RNA sequencing of microglia and monocyte-derived macrophages from mice with experimental autoimmune encephalomyelitis illustrates a changing phenotype with disease course. J Neuroimmunol. (2014) 277:26–38. 10.1016/j.jneuroim.2014.09.014 25270668

[141] El-Behi M Ciric B Dai H Yan Y Cullimore M Safavi F. The encephalitogenicity of T(H)17 cells is dependent on IL-1- and IL-23-induced production of the cytokine GM-CSF. Nat Immunol. (2011) 12:568–75. 10.1038/ni.2031 21516111 PMC3116521

[142] Piccio L Buonsanti C Mariani M Cella M Gilfillan S Cross AH. Blockade of TREM-2 exacerbates experimental autoimmune encephalomyelitis. Eur J Immunol. (2007) 37:1290–301. 10.1002/eji.200636837 17407101

[143] Olah M Amor S Brouwer N Vinet J Eggen B Biber K. Identification of a microglia phenotype supportive of remyelination. Glia. (2012) 60:306–21. 10.1002/glia.21266 22072381

[144] Poliani PL Wang Y Fontana E Robinette ML Yamanishi Y Gilfillan S. TREM2 sustains microglial expansion during aging and response to demyelination. J Clin Invest. (2015) 125:2161–70. 10.1172/JCI77983 25893602 PMC4463196

[145] Masuda T Sankowski R Staszewski O Böttcher C Amann L Scheiwe C. Spatial and temporal heterogeneity of mouse and human microglia at single-cell resolution. Nature. (2019) 566:388–92. 10.1038/s41586-019-0924-x 30760929

[146] Laflamme N Cisbani G Préfontaine P Srour Y Bernier J St-Pierre M-K. mCSF-Induced microglial activation prevents myelin loss and promotes its repair in a mouse model of multiple sclerosis. Front Cell Neurosci. (2018) 12:178. 10.3389/fncel.2018.00178 30018535 PMC6037698

[147] Cantoni C Bollman B Licastro D Xie M Mikesell R Schmidt R. TREM2 regulates microglial cell activation in response to demyelination *in vivo*. Acta Neuropathol. (2015) 129:429–47. 10.1007/s00401-015-1388-1 25631124 PMC4667728

[148] Lloyd AF Davies CL Holloway RK Labrak Y Ireland G Carradori D. Central nervous system regeneration is driven by microglia necroptosis and repopulation. Nat Neurosci. (2019) 22:1046–52. 10.1038/s41593-019-0418-z 31182869 PMC6597360

[149] Elliott R Li F Dragomir I Chua MMW Gregory BD Weiss SR. Analysis of the host transcriptome from demyelinating spinal cord of murine coronavirus-infected mice. PLoS ONE. (2013) 8:e75346. 10.1371/journal.pone.0075346 24058676 PMC3776850

[150] Charles NA Holland EC Gilbertson R Glass R Kettenmann H. The brain tumor microenvironment. Glia. (2011) 59:1169–80. 10.1002/glia.21136 21446047

[151] Li W Graeber MB. The molecular profile of microglia under the influence of glioma. Neuro-Oncol. (2012) 14:958–78. 10.1093/neuonc/nos116 22573310 PMC3408253

[152] Szulzewsky F Pelz A Feng X Synowitz M Markovic D Langmann T. Glioma-Associated microglia/macrophages display an expression profile different from M1 and M2 polarization and highly express Gpnmb and spp1. PLoS ONE. (2015) 10:e0116644. 10.1371/journal.pone.0116644 25658639 PMC4320099

[153] Kucukural A Yukselen O Ozata DM Moore MJ Garber M. DEBrowser: interactive differential expression analysis and visualization tool for count data. BMC Genomics. (2019) 20:6. 10.1186/s12864-018-5362-x 30611200 PMC6321710

[154] Dubbelaar ML Kracht L Eggen BJL Boddeke EWGM. The kaleidoscope of microglial phenotypes. Front Immunol. (2018) 9:1753. 10.3389/fimmu.2018.01753 30108586 PMC6079257

[155] Gonzalez-Pena D Nixon SE O'Connor JC Southey BR Lawson MA McCusker RH. Microglia transcriptome changes in a model of depressive behavior after immune challenge. PLoS ONE. (2016) 11:e0150858. 10.1371/journal.pone.0150858 26959683 PMC4784788

[156] Sousa C Golebiewska A Poovathingal SK Kaoma T Pires-Afonso Y Martina S. Single-cell transcriptomics reveals distinct inflammation-induced microglia signatures. EMBO Rep. (2018) 19:e46171. 10.15252/embr.201846171 30206190 PMC6216255

[157] Butovsky O Bukshpan S Kunis G Jung S Schwartz M. Microglia can be induced by IFN-γ or IL-4 to express neural or dendritic-like markers. Mol Cell Neurosci. (2007) 35:490–500. 10.1016/j.mcn.2007.04.009 17560122

