# Supplementary material for: Protective Microglial Subset in Development, Aging, and Disease: Lessons From Transcriptomic Studies
Source: Front Immunol. 2020 Apr 3;11:430. doi: 10.3389/fimmu.2020.00430 (PMC7147523; doi:10.3389/fimmu.2020.00430)
Supplement: Supplementary file 1 [file Table_1.docx]

Supplementary Material

# Supplementary Table. Function for the proteins associated to the core signature genes.

| Gene name | Protein name | Protein function | Global roles | Roles in the CNS | References |
| --- | --- | --- | --- | --- | --- |
| Ank | Ankyrin-1 | Adaptor protein | - Adhesion molecules binding - Serpin binding - CD44 interaction - Participation in cell proliferation, mobility, activation - Membrane proteins attachment | - Production at dendritic spines, modulation of LTD - Expression by microglia in AD - Hypermethylated in AD, PD and HD | (1,2) |
| Anxa5 | Annexin A5 | Lipid-binding protein | - Phospholipids binding - Integrin and heparin binding - Inhibition of phospholipase A - Phagocytosis of apoptotic cells (involved in antigen processing and presentation) - Suppression of proinflammatory cytokine secretion - Inhibition of DCs - Involved in cholesterol metabolism - Possible involvement in membrane repair | - Not expressed in neurons - Increased in AD - Increased in glioma | (3–7) |
| Aplp2 | Amyloid precursor-like protein 2 | Protease inhibitor | - Heparin binding - Modulation of insulin homeostasis and IGF transport - Binding and endocytosis of MHCI molecules | - Presence in proliferative zones in development - Involvement in axonal guidance and synaptic plasticity - Modulation of demyelination/remyelination - Increased in glioblastooma | (8–12) |
| Atp1a3 | Na(+)/K(+) ATPase alpha-3 subunit | Translocase | - ATP-dependent transport of Na+ and K+ across the plasma membrane - Heparan sulfate binding leading to inhibition | - Suposedly restricted to neurons - Association with Aβ, α-synuclein and SOD1 assemblies - Mutations lead to rapid onset dystonia parkinsonism, hemiplegia, cerebellar ataxia, areflexia, pes cavus, optic atrophy, sensorineural hearing loss, early infantile epileptic encephalopathy, autism spectrum disorders, schizophrenia | (13) |
| Clec7a | C-type lectin domain family 7 member | Receptor | - Galectin, zymosan and β-glucan binding - Modulation of cytokine and ROS production by macrophages and DCs - Modulation of innate immune memory, T cell activation and proliferation, and B cell responses - Regulation of autophagy, phagocytosis and the respiratory burst - Anti-tumor properties | - No induction of cytokine production in microglia in response to β-glucan - Detrimental when expressed by microglia in the context of SCI - Promotion of axon regeneration in the optic nerve - Possible involvement in autism spectrum disorders | (14–20) |
| Colec12 | Collectin-12 | Receptor (scavenger) | - Carbohydrates, oxLDL and C-type lectins binding - Regulation of LDL cholesterol in the circulation - Activation of innate immune responses and complement - Involvement in developmental processes | - Facilitation of Aβ clearance by microglia in AD - Increased in AD patients - Increased in myelin-containing phagocytes in MS lesions | (21,22) |
| Csf1 | Colony stimulating factor 1 | Growth factor / Cytokine | - Regulation of survival, proliferation and differentiation of macrophages and monocytes - Modulation of cytokine production in innate immune responses and inflammation - Promotion of reorganization of actin cytoskeleton for cell adhesion and migration - Promotion of lipoprotein clearance | - Expressed by neurons and microglia - Regulates microglia development, particularly in the white matter - Increases DAP12 and decreases antigen presentation in microglia - Decreases macrophage inflammatory phenotype - Promotion of myelin and Aβ clearance by microglia - Induction of IGFBP1 for increased angiogenesis in glioblastoma - Regulation of microglial proliferation in ALS - Increased in glioblastoma | (23–27) |
| Ephx1 | Epoxide hydrolase 1 | Hydrolase | - Converting epoxides in diols for detoxification or bioactivation | - Expression in neurons and astrocytes - Contribution to cerebral metabolism - Increased in glioma - Increased in AD | (28) |
| Fabp5 | Fatty acid binding protein 5 | Lipid-binding protein | - Fatty acid uptake, transport and metabolism - Expression in macrophages for cytokine production and mediation of cellular stress responses - Interaction with PPAR nuclear receptors | - Expression in the perinatal brain - Involvement in motor neuron and astrocyte differentiation - Possible involvement in neurite outgrowth, axon development ad neural cell regeneration - Possible involvement in neurogenesis - Increased in excitotoxic lesions - Increased in peripheral nerve injury - Increased in ischemia | (29–31) |
| Fam20c | Extracellular serine/threonine protein kinase FAM20C | Kinase | - Phosphorylation of fibrinogen - Phosphoryplation of casein - Phosphorylation of osteopontin (SPP1) - Regulation of IGF transport and uptake by IGFBP | - Phosphorylates APP and APOE - Activated by sphingosine and fingolimod | (32–36) |
| Gm1673 | Neuropeptide-like protein C4orf48 homolog | Neuropeptide |  | - Expression in development and adulthood in cortex - Mutation leads to Wolf-Hirschhorn syndrome | (37) |
| Gpnmb | Transmembrane glycoprotein NMB | Glycoprotein / Chemoattractant | - Heparin and integrin binding - Interaction with CD44 - Involvement in motility and angiogenesis through Wnt signaling - Negative regulation of T cell activation via syndecan binding - Involvement in lysosome function | - Contribution to memory - Protection following ischemia - Protection in ALS - Participates in glioblastoma growth through Wnt signaling and NA+/K+ ATPase - Increased in glioblastoma, - Increased in AD (in microglia) - Increased in PD - Increased in ALS - Increased in MS lesions (in foamy macrophages) | (38–42) |
| Hpse | Heparanase | Heparanase | - Heparan sulfate cleavage leading to extracellular matrix integrity reduction - Regulation of signaling pathways - Facilitation of cell migration - Involvement in syndecan shedding - Participation in tumor invasion | - Expression in brain development - Involvement in microglial migration - Restriction of microglial inflammatory response - Dual roles in EAE and MS - Dual roles in AD - Delays prion disease onset - Promotes glioma progression - Increased in AD - Increased in glioma - Increased in stroke | (43–47) |
| Igf1 | Insulin-like growth factor 1 | Growth factor / Hormone | - Signaling downstream of growth hormone - Involvement in cell growth and maturation - Activation of anabolic processes - Association with decreased lifespan - Interaction with integrins (including the SPP1 receptor) | - Promotion of neuron survival and primary myelination during development by microglia expression - Presence in neurogenic niches - Involvement in protection against cellular injury, neurogenesis, angiogenesis and amyloid clearance - Dual roles in AD | (48–50) |
| Itgax | Integrin alpha-x | Integrin / Receptor | - Binds to CD18 to make up complement receptor 4 (for iC3b) - Fibrinogen binding - Mediation of cellular interactions during inflammatory responses - Mediation of cell recruitment - Regulation of cytoskeleton rearrangement - Regulation of activation and proliferation of leucocytes | - Reviewed here | (51) |
| Lilrb4 | Leucocyte immunoglobulin-like receptor subfamily B member 4 | Receptor  (Ig superfamily) | - MHCI binding - Induced by IFNβ - Promotion of tolerance by immune response downregulation | - Involvement in immune responsiveness in MS - Increased in aging - Increased in MS lesions | (52–54) |
| Lpl | Lipoprotein lipase | Hydrolase | - Involvement in blood lipoprotein triglyceride catabolism - Activated by Apolipoprotein C - Participation in fatty acid transport - Participation in lipoprotein formation (LDL, VLDL, HDL) - Possible involvement in cellular interactions - Interaction with heparan proteoglycans - Interaction with APOE | - Regulation of neuronal survival and proliferation - Promotion of foam cell formation - Reduction in microglia leads to decreased lipid uptake, shift in mitochondria activity and decreased immune reactivity - Participation in myelin clearance - Increased in AD - Increased in glioma | (55,56) |
| Nceh1 | Neutral cholesterol ester hydrolase 1 | Hydrolase | - Involvement in reverse cholesterol transport - Platelet activating factor biosynthesis - Lipophospholipid signaling - Detoxification - Cholesterol esters lipolysis | - Involved in formation of foam cells | (57–59) |
| Plaur | Urokinase plasminogen activator surface receptor | Receptor  (Ig superfamily) | - Integrin, LRP1, EGR and PDGFRβ binding as coreceptors - Participation in cell migration, proliferation and survival - Induction of actin cytoskeleton reorganization | - High expression during development and disease, contraction in adulthood - Involvement in synaptic repair and axonal recovery - Presence in injury, MS and prion disease - Increased in epilepsy - Increased in AD - Increased in glioma | (60,61) |
| Pld3 | Phospholipase D3 | Hydrolase | - Unclear functions (Possible function in hydrolysis of membrane phospholipids) | - Expressed in brain development - Possible involvement in APP processing - Risk variant for AD | (62,63) |
| Plin2 | Perilipin-2 | Lipid binding protein | - Involvement in formation of lipid droplets - Interaction with PPAR nuclear reveptors | - Increased in injury (in microglia) | (64–66) |
| Spp1 | Osteopontin | Cytokine | - Integrin binding (particularly CD11c/CD18) - CD44 interaction - Involvement in cellular motility, adhesion and survival - Regulation of developmental processes, tissue remodeling and immune function - Possible inhibition of apoptosis - Possible modulation of T cell activation - Regulated by IFNβ | - Involvement in neural precursor proliferation - Involvement in memory and myelination in development - Participation in synaptic plasticity, reinnervation, axonal growth, synapse reorganization and functional recovery following injury - Enhances immunosuppression - Increased in AD - Increased in MS - Increased in glioblastoma - Increased in NMDAR encephalitis - Decreased in PD | (67–70) |

**References**

1. Smith KR, Penzes P. Ankyrins: Roles in synaptic biology and pathology. *Mol Cell Neurosci* (2018) **91**:131–139. doi:10.1016/j.mcn.2018.04.010

2. Smith AR, Smith RG, Burrage J, Troakes C, Al-Sarraj S, Kalaria RN, Sloan C, Robinson AC, Mill J, Lunnon K. A cross-brain regions study of ANK1 DNA methylation in different neurodegenerative diseases. *Neurobiol Aging* (2019) **74**:70–76. doi:10.1016/j.neurobiolaging.2018.09.024

3. Bouter A, Carmeille R, Gounou C, Bouvet F, Degrelle SA, Evain-Brion D, Brisson AR. Review: Annexin-A5 and cell membrane repair. *Placenta* (2015) **36**:S43–S49. doi:10.1016/j.placenta.2015.01.193

4. Pedrero-Prieto CM, Flores-Cuadrado A, Saiz-Sánchez D, Úbeda-Bañón I, Frontiñán-Rubio J, Alcaín FJ, Mateos-Hernández L, de la Fuente J, Durán-Prado M, Villar M, et al. Human amyloid-β enriched extracts: evaluation of in vitro and in vivo internalization and molecular characterization. *Alzheimers Res Ther* (2019) **11**: doi:10.1186/s13195-019-0513-0

5. Peng B, Guo C, Guan H, Liu S, Sun M-Z. Annexin A5 as a potential marker in tumors. *Clin Chim Acta Int J Clin Chem* (2014) **427**:42–48. doi:10.1016/j.cca.2013.09.048

6. Rentero C, Blanco-Muñoz P, Meneses-Salas E, Grewal T, Enrich C. Annexins—Coordinators of Cholesterol Homeostasis in Endocytic Pathways. *Int J Mol Sci* (2018) **19**:1444. doi:10.3390/ijms19051444

7. Weyd H. More than just innate affairs – on the role of annexins in adaptive immunity. *Biol Chem* (2016) **397**:1017–1029. doi:10.1515/hsz-2016-0191

8. Chen Y, Wang H, Tan C, Yan Y, Shen J, Huang Q, Xu T, Lin J, Chen J. Expression of amyloid precursor-like protein 2 (APLP2) in glioblastoma is associated with patient prognosis. *Folia Neuropathol* (2018) **56**:30–38. doi:10.5114/fn.2018.74657

9. Müller UC, Deller T, Korte M. Not just amyloid: physiological functions of the amyloid precursor protein family. *Nat Rev Neurosci* (2017) **18**:281–298. doi:10.1038/nrn.2017.29

10. Needham BE, Wlodek ME, Ciccotosto GD, Fam BC, Masters CL, Proietto J, Andrikopoulos S, Cappai R. Identification of the Alzheimer’s disease amyloid precursor protein (APP) and its homologue APLP2 as essential modulators of glucose and insulin homeostasis and growth. *J Pathol* (2008) **215**:155–163. doi:10.1002/path.2343

11. Truong PH, Ciccotosto GD, Merson TD, Spoerri L, Chuei MJ, Ayers M, Xing YL, Emery B, Cappai R. Amyloid precursor protein and amyloid precursor-like protein 2 have distinct roles in modulating myelination, demyelination, and remyelination of axons. *Glia* (2019) **67**:525–538. doi:10.1002/glia.23561

12. Tuli A, Sharma M, McIlhaney MM, Talmadge JE, Naslavsky N, Caplan S, Solheim JC. Amyloid Precursor-Like Protein 2 Increases the Endocytosis, Instability, and Turnover of the H2-Kd MHC Class I Molecule. *J Immunol* (2008) **181**:1978–1987. doi:10.4049/jimmunol.181.3.1978

13. Shrivastava AN, Triller A, Melki R. Cell biology and dynamics of Neuronal Na+/K+-ATPase in health and diseases. *Neuropharmacology* (2018)107461. doi:10.1016/j.neuropharm.2018.12.008

14. Baldwin KT, Carbajal KS, Segal BM, Giger RJ. Neuroinflammation triggered by β-glucan/dectin-1 signaling enables CNS axon regeneration. *Proc Natl Acad Sci U S A* (2015) **112**:2581–2586. doi:10.1073/pnas.1423221112

15. Bennabi M, Delorme R, Oliveira J, Fortier C, Lajnef M, Boukouaci W, Feugeas J-P, Marzais F, Gaman A, Charron D, et al. Dectin-1 Polymorphism: A Genetic Disease Specifier in Autism Spectrum Disorders? *PLOS ONE* (2015) **10**:e0137339. doi:10.1371/journal.pone.0137339

16. Chiba S, Ikushima H, Ueki H, Yanai H, Kimura Y, Hangai S, Nishio J, Negishi H, Tamura T, Saijo S, et al. Recognition of tumor cells by Dectin-1 orchestrates innate immune cells for anti-tumor responses. *eLife* (2014) **3**:e04177. doi:10.7554/eLife.04177

17. Chiffoleau E. C-Type Lectin-Like Receptors As Emerging Orchestrators of Sterile Inflammation Represent Potential Therapeutic Targets. *Front Immunol* (2018) **9**: doi:10.3389/fimmu.2018.00227

18. Dambuza IM, Brown GD. C-type lectins in immunity: recent developments. *Curr Opin Immunol* (2015) **32**:21–27. doi:10.1016/j.coi.2014.12.002

19. Gensel JC, Wang Y, Guan Z, Beckwith KA, Braun KJ, Wei P, McTigue DM, Popovich PG. Toll-Like Receptors and Dectin-1, a C-Type Lectin Receptor, Trigger Divergent Functions in CNS Macrophages. *J Neurosci* (2015) **35**:9966–9976. doi:10.1523/JNEUROSCI.0337-15.2015

20. Tone K, Stappers MHT, Willment JA, Brown GD. C-type lectin receptors of the Dectin-1 cluster: Physiological roles and involvement in disease. *Eur J Immunol* **0**: doi:10.1002/eji.201847536

21. Bogie JFJ, Mailleux J, Wouters E, Jorissen W, Grajchen E, Vanmol J, Wouters K, Hellings N, van Horssen J, Vanmierlo T, et al. Scavenger receptor collectin placenta 1 is a novel receptor involved in the uptake of myelin by phagocytes. *Sci Rep* (2017) **7**:44794. doi:10.1038/srep44794

22. Hansen SWK, Ohtani K, Roy N, Wakamiya N. The collectins CL-L1, CL-K1 and CL-P1, and their roles in complement and innate immunity. *Immunobiology* (2016) **221**:1058–1067. doi:10.1016/j.imbio.2016.05.012

23. Chitu V, Stanley ER. “Chapter Seven - Regulation of Embryonic and Postnatal Development by the CSF-1 Receptor,” in *Current Topics in Developmental Biology* Protein Kinases in Development and Disease., ed. A. Jenny (Academic Press), 229–275. doi:10.1016/bs.ctdb.2016.10.004

24. Chitu V, Gokhan Ş, Nandi S, Mehler MF, Stanley ER. Emerging Roles for CSF-1 Receptor and its Ligands in the Nervous System. *Trends Neurosci* (2016) **39**:378–393. doi:10.1016/j.tins.2016.03.005

25. Easley-Neal C, Foreman O, Sharma N, Zarrin AA, Weimer RM. CSF1R Ligands IL-34 and CSF1 Are Differentially Required for Microglia Development and Maintenance in White and Gray Matter Brain Regions. *Front Immunol* (2019) **10**: doi:10.3389/fimmu.2019.02199

26. Martínez-Muriana A, Mancuso R, Francos-Quijorna I, Olmos-Alonso A, Osta R, Perry VH, Navarro X, Gomez-Nicola D, López-Vales R. CSF1R blockade slows the progression of amyotrophic lateral sclerosis by reducing microgliosis and invasion of macrophages into peripheral nerves. *Sci Rep* (2016) **6**:25663. doi:10.1038/srep25663

27. Nijaguna MB, Patil V, Urbach S, Shwetha SD, Sravani K, Hegde AS, Chandramouli BA, Arivazhagan A, Marin P, Santosh V, et al. Glioblastoma-derived Macrophage Colony-stimulating Factor (MCSF) Induces Microglial Release of Insulin-like Growth Factor-binding Protein 1 (IGFBP1) to Promote Angiogenesis. *J Biol Chem* (2015) **290**:23401–23415. doi:10.1074/jbc.M115.664037

28. Václavíková R, Hughes DJ, Souček P. Microsomal epoxide hydrolase 1 (EPHX1): Gene, structure, function, and role in human disease. *Gene* (2015) **571**:1–8. doi:10.1016/j.gene.2015.07.071

29. Matsumata M, Inada H, Osumi N. Fatty acid binding proteins and the nervous system: Their impact on mental conditions. *Neurosci Res* (2016) **102**:47–55. doi:10.1016/j.neures.2014.08.012

30. Owada Y. Fatty Acid Binding Protein: Localization and Functional Significance in the Brain. *Tohoku J Exp Med* (2008) **214**:213–220. doi:10.1620/tjem.214.213

31. Storch J, Thumser AE. Tissue-specific Functions in the Fatty Acid-binding Protein Family. *J Biol Chem* (2010) **285**:32679–32683. doi:10.1074/jbc.R110.135210

32. Cozza G, Salvi M, Banerjee S, Tibaldi E, Tagliabracci VS, Dixon JE, Pinna LA. A new role for sphingosine: Up-regulation of Fam20C, the genuine casein kinase that phosphorylates secreted proteins. *Biochim Biophys Acta BBA - Proteins Proteomics* (2015) **1854**:1718–1726. doi:10.1016/j.bbapap.2015.04.023

33. Park BC, Reese M, Tagliabracci VS. Thinking outside of the cell: Secreted protein kinases in bacteria, parasites, and mammals. *IUBMB Life* (2019) **71**:749–759. doi:10.1002/iub.2040

34. Sreelatha A, Kinch LN, Tagliabracci VS. The secretory pathway kinases. *Biochim Biophys Acta BBA - Proteins Proteomics* (2015) **1854**:1687–1693. doi:10.1016/j.bbapap.2015.03.015

35. Tagliabracci VS, Pinna LA, Dixon JE. Secreted protein kinases. *Trends Biochem Sci* (2013) **38**:121. doi:10.1016/j.tibs.2012.11.008

36. Tagliabracci VS, Wiley SE, Guo X, Kinch LN, Durrant E, Wen J, Xiao J, Cui J, Nguyen KB, Engel JL, et al. A Single Kinase Generates the Majority of the Secreted Phosphoproteome. *Cell* (2015) **161**:1619–1632. doi:10.1016/j.cell.2015.05.028

37. Endele S, Nelkenbrecher C, Bördlein A, Schlickum S, Winterpacht A. C4ORF48, a gene from the Wolf-Hirschhorn syndrome critical region, encodes a putative neuropeptide and is expressed during neocortex and cerebellar development. *Neurogenetics* (2011) **12**:155–163. doi:10.1007/s10048-011-0275-8

38. Budge KM, Neal ML, Richardson JR, Safadi FF. Glycoprotein NMB: an Emerging Role in Neurodegenerative Disease. *Mol Neurobiol* (2018) **55**:5167–5176. doi:10.1007/s12035-017-0707-z

39. Chung J-S, Bonkobara M, Tomihari M, Cruz PD, Ariizumi K. The DC-HIL/syndecan-4 pathway inhibits human allogeneic T-cell responses. *Eur J Immunol* (2009) **39**:965–974. doi:10.1002/eji.200838990

40. Hendrickx DAE, van Scheppingen J, van der Poel M, Bossers K, Schuurman KG, van Eden CG, Hol EM, Hamann J, Huitinga I. Gene Expression Profiling of Multiple Sclerosis Pathology Identifies Early Patterns of Demyelination Surrounding Chronic Active Lesions. *Front Immunol* (2017) **8**: doi:10.3389/fimmu.2017.01810

41. van der Lienden MJC, Gaspar P, Boot R, Aerts JMFG, van Eijk M. Glycoprotein Non-Metastatic Protein B: An Emerging Biomarker for Lysosomal Dysfunction in Macrophages. *Int J Mol Sci* (2018) **20**: doi:10.3390/ijms20010066

42. Ono Y, Chiba S, Yano H, Nakayama N, Saio M, Tsuruma K, Shimazawa M, Iwama T, Hara H. Glycoprotein nonmetastatic melanoma protein B (GPNMB) promotes the progression of brain glioblastoma via Na+/K+-ATPase. *Biochem Biophys Res Commun* (2016) **481**:7–12. doi:10.1016/j.bbrc.2016.11.034

43. Changyaleket B, Deliu Z, Chignalia AZ, Feinstein DL. Heparanase: Potential roles in multiple sclerosis. *J Neuroimmunol* (2017) **310**:72–81. doi:10.1016/j.jneuroim.2017.07.001

44. García B, Martín C, García-Suárez O, Muñiz-Alonso B, Ordiales H, Fernández-Menéndez S, Santos-Juanes J, Lorente-Gea L, Castañón S, Vicente-Etxenausia I, et al. Upregulated Expression of Heparanase and Heparanase 2 in the Brains of Alzheimer’s Disease. *J Alzheimers Dis* (2017) **58**:185–192. doi:10.3233/JAD-161298

45. Kovalchuk Ben-Zaken O, Nissan I, Tzaban S, Taraboulos A, Zcharia E, Matzger S, Shafat I, Vlodavsky I, Tal Y. Transgenic over-expression of mammalian heparanase delays prion disease onset and progression. *Biochem Biophys Res Commun* (2015) **464**:698–704. doi:10.1016/j.bbrc.2015.06.170

46. O’Callaghan P, Zhang X, Li J-P. Heparan Sulfate Proteoglycans as Relays of Neuroinflammation. *J Histochem Cytochem* (2018) **66**:305–319. doi:10.1369/0022155417742147

47. Tran VM, Wade A, McKinney A, Chen K, Lindberg OR, Engler JR, Persson AI, Phillips JJ. Heparan Sulfate Glycosaminoglycans in Glioblastoma Promote Tumor Invasion. *Mol Cancer Res* (2017) **15**:1623–1633. doi:10.1158/1541-7786.MCR-17-0352

48. Dyer AH, Vahdatpour C, Sanfeliu A, Tropea D. The role of Insulin-Like Growth Factor 1 (IGF-1) in brain development, maturation and neuroplasticity. *Neuroscience* (2016) **325**:89–99. doi:10.1016/j.neuroscience.2016.03.056

49. Werner H, LeRoith D. Insulin and insulin-like growth factor receptors in the brain: Physiological and pathological aspects. *Eur Neuropsychopharmacol* (2014) **24**:1947–1953. doi:10.1016/j.euroneuro.2014.01.020

50. Wrigley S, Arafa D, Tropea D. Insulin-Like Growth Factor 1: At the Crossroads of Brain Development and Aging. *Front Cell Neurosci* (2017) **11**: doi:10.3389/fncel.2017.00014

51. Schittenhelm L, Hilkens CM, Morrison VL. β2 Integrins As Regulators of Dendritic Cell, Monocyte, and Macrophage Function. *Front Immunol* (2017) **8**: doi:10.3389/fimmu.2017.01866

52. Jensen MA, Yanowitch RN, Reder AT, White DM, Arnason BGW. Immunoglobulin-like transcript 3, an inhibitor of T cell activation, is reduced on blood monocytes during multiple sclerosis relapses and is induced by interferon beta-1b. *Mult Scler Houndmills Basingstoke Engl* (2010) **16**:30–38. doi:10.1177/1352458509352794

53. Vlad G, Chang C-C, Colovai AI, Vasilescu ER, Cortesini R, Suciu-Foca N. Membrane and Soluble ILT3 Are Critical to the Generation of T Suppressor Cells and Induction of Immunological Tolerance. *Int Rev Immunol* (2010) **29**:119–132. doi:10.3109/08830180903281185

54. Waschbisch A, Sanderson N, Krumbholz M, Vlad G, Theil D, Schwab S, Mäurer M, Derfuss T. Interferon beta and vitamin D synergize to induce immunoregulatory receptors on peripheral blood monocytes of multiple sclerosis patients. *PloS One* (2014) **9**:e115488. doi:10.1371/journal.pone.0115488

55. Chang C. Lipoprotein lipase: new roles for an ‘old’ enzyme. *Curr Opin Clin Nutr Metab Care* (2019) **22**:111–115. doi:10.1097/MCO.0000000000000536

56. Mead JR, Irvine SA, Ramji DP. Lipoprotein lipase: structure, function, regulation, and role in disease. *J Mol Med* (2002) **80**:753–769. doi:10.1007/s00109-002-0384-9

57. Ghosh S. Early steps in reverse cholesterol transport: cholesteryl ester hydrolase and other hydrolases. *Curr Opin Endocrinol Diabetes Obes* (2012) **19**:136. doi:10.1097/MED.0b013e3283507836

58. Igarashi Masaki, Osuga Jun-ichi, Uozaki Hiroshi, Sekiya Motohiro, Nagashima Shuichi, Takahashi Manabu, Takase Satoru, Takanashi Mikio, Li Yongxue, Ohta Keisuke, et al. The Critical Role of Neutral Cholesterol Ester Hydrolase 1 in Cholesterol Removal From Human Macrophages. *Circ Res* (2010) **107**:1387–1395. doi:10.1161/CIRCRESAHA.110.226613

59. Quiroga AD, Lehner R. Role of endoplasmic reticulum neutral lipid hydrolases. *Trends Endocrinol Metab* (2011) **22**:218–225. doi:10.1016/j.tem.2011.03.003

60. Blasi F, Carmeliet P. uPAR: a versatile signalling orchestrator. *Nat Rev Mol Cell Biol* (2002) **3**:932–943. doi:10.1038/nrm977

61. Yepes M. Urokinase-type plasminogen activator is a modulator of synaptic plasticity in the central nervous system: implications for neurorepair in the ischemic brain. *Neural Regen Res* (2020) **15**:620–624. doi:10.4103/1673-5374.266904

62. Blanco-Luquin I, Altuna M, Sánchez-Ruiz de Gordoa J, Urdánoz-Casado A, Roldán M, Cámara M, Zelaya V, Erro ME, Echavarri C, Mendioroz M. PLD3 epigenetic changes in the hippocampus of Alzheimer’s disease. *Clin Epigenetics* (2018) **10**:116. doi:10.1186/s13148-018-0547-3

63. Gonzalez AC, Schweizer M, Jagdmann S, Bernreuther C, Reinheckel T, Saftig P, Damme M. Unconventional Trafficking of Mammalian Phospholipase D3 to Lysosomes. *Cell Rep* (2018) **22**:1040–1053. doi:10.1016/j.celrep.2017.12.100

64. Chali F, Milior G, Marty S, Morin-Brureau M, Le Duigou C, Savary E, Blugeon C, Jourdren L, Miles R. Lipid markers and related transcripts during excitotoxic neurodegeneration in kainate-treated mice. *Eur J Neurosci* (2019) **50**:1759–1778. doi:10.1111/ejn.14375

65. Kimmel AR, Sztalryd C. The Perilipins: Major Cytosolic Lipid Droplet–Associated Proteins and Their Roles in Cellular Lipid Storage, Mobilization, and Systemic Homeostasis. *Annu Rev Nutr* (2016) **36**:471–509. doi:10.1146/annurev-nutr-071813-105410

66. Sztalryd C, Brasaemle DL. The perilipin family of lipid droplet proteins: Gatekeepers of intracellular lipolysis. *Biochim Biophys Acta BBA - Mol Cell Biol Lipids* (2017) **1862**:1221–1232. doi:10.1016/j.bbalip.2017.07.009

67. Carecchio M, Comi C. The Role of Osteopontin in Neurodegenerative Diseases. *J Alzheimers Dis* (2011) **25**:179–185. doi:10.3233/JAD-2011-102151

68. Del Prete A, Scutera S, Sozzani S, Musso T. Role of osteopontin in dendritic cell shaping of immune responses. *Cytokine Growth Factor Rev* (2019) doi:10.1016/j.cytogfr.2019.05.004

69. Icer MA, Gezmen-Karadag M. The multiple functions and mechanisms of osteopontin. *Clin Biochem* (2018) **59**:17–24. doi:10.1016/j.clinbiochem.2018.07.003

70. Yu H, Liu X, Zhong Y. The Effect of Osteopontin on Microglia. *BioMed Res Int* (2017) doi:10.1155/2017/1879437

**
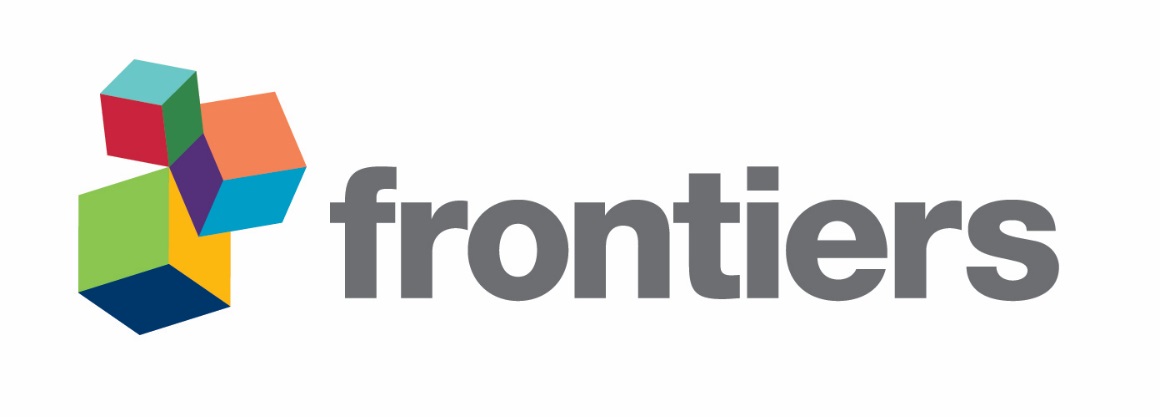
**
